# Maximizing Engagement, Trust, and Clinical Benefit of AI-Generated Recovery Support Messages for Alcohol Use Disorder: Protocol for an Optimization Study

**DOI:** 10.2196/81697

**Published:** 2025-11-07

**Authors:** Kendra Wyant, Sarah J Sant'Ana, Claire E Punturieri, Jiachen Yu, Gaylen E Fronk, C Michael Maggard, Christopher Janssen, Susan E Wanta, Rachel Kornfield, Lyn M van Swol, John J Curtin

**Affiliations:** 1 Department of Psychology University of Wisconsin-Madison Madison, WI United States; 2 Department of Psychiatry and Behavioral Sciences Medical University of South Carolina Charleston, SC United States; 3 Department of Electrical and Computer Engineering University of Wisconsin-Madison Madison, WI United States; 4 Department of Preventive Medicine Northwestern University Chicago, IL United States; 5 Department of Communication Arts University of Wisconsin-Madison Madison, WI United States

**Keywords:** substance use disorders, precision mental health, continuing care, relapse prevention, MOST, Multiphase Optimization Strategy

## Abstract

**Background:**

Successful recovery from alcohol use disorder requires long-term lapse risk monitoring. Self-monitoring is difficult, given the dynamic, complex interplay of the many risk factors over time. An automated recovery monitoring support system embedded with a machine learning lapse prediction model could improve sustained, adaptive, and personalized self-monitoring by delivering daily support messages.

**Objective:**

We propose to optimize the components included in daily support messages to increase engagement with a recovery monitoring support system.

**Methods:**

The participants will include 304 US adults with moderate to severe alcohol use disorder. Participants will complete daily surveys and provide geolocation data for 17 weeks. Participants will receive daily support messages, starting in week 2, that convey a combination of individualized information from a lapse prediction model. Manipulated message components include (1) lapse probability and lapse probability change, (2) an important model feature, (3) a risk-relevant recommendation, and (4) message personalization on tone preference.

**Results:**

The National Institute on Alcohol Abuse and Alcoholism funded this project (R01AA031762) on August 9, 2024, with a funding period from August 20, 2024, to July 31, 2029. The institutional research board of the University of Wisconsin-Madison Health Sciences approved this project (IRB #2024-0869). Enrollment will begin in December 2025.

**Conclusions:**

Message components that either increase engagement or improve clinical outcomes will be recommended for use in future recovery monitoring support systems and digital therapeutics.

**International Registered Report Identifier (IRRID):**

PRR1-10.2196/81697

## Introduction

### Alcohol Use Disorder

Alcohol use disorder is highly prevalent and costly. Over 30 million adults in the United States had an active alcohol use disorder in 2021, and 23.3% reported engaging in past-month binge drinking [1]. Alcohol ranks as the third leading preventable cause of death in the United States, accounting for approximately 140,000 fatalities [2,3] and economic costs that exceed US $249 billion annually [4].

### Relapse Prevention

Alcohol use disorder is a chronic condition. Relapse rates following initial treatment are high [5,6]. Lapses (ie, single instances of goal-inconsistent alcohol use) are a necessary precursor for relapse (ie, full return to harmful levels of alcohol use). As a result, preventing lapses and guiding behavior change immediately following a lapse are key goals in both acute and continuing care alcohol use disorder treatment.

Marlatt and Gordon’s seminal relapse prevention model [7,8] provides the backbone for contemporary, clinician-delivered interventions for alcohol use disorder (eg, cognitive behavioral therapy [9,10] and mindfulness-based relapse prevention [11]) that have the highest level of empirical support [12-14]. The relapse prevention model provides a detailed framework to understand how emotions, events, and situations can lead to lapses and relapse back to alcohol use. This framework includes both distal and proximal precipitants of lapse such as lifestyle imbalances, craving, high-risk situations, stressors, negative affect, self-efficacy or confidence, and abstinence violation effects. The model’s influence is clearly seen in numerous, efficacious intervention modules and supports that are included in cognitive behavioral and mindfulness-based interventions (eg, coping skills training, lapse management, urge surfing, stimulus control techniques, and positive lifestyle changes).

The relapse prevention model highlights that relapse is a complex, nonlinear function of many risk and protective factors that combine and interact to affect relapse timing and severity [15-19]. Many of these factors are transient, leading to fluctuating relapse risk. Urges, mood, lifestyle imbalances, self-efficacy, and motivation all vary over time. Social networks evolve to be more protective or risky. High-risk situations can occur at any time.

Much like other chronic conditions, clinical observation and research suggest that successful recovery from alcohol use disorder requires lifelong monitoring [15,17,19-21]. However, individual (eg, clinician bandwidth) and systemic (eg, overburdened health care system) resources are insufficient for long-term clinician-guided care. Additionally, self-monitoring is difficult, given the dynamic, complex interplay of many risk factors over time.

### Recovery Monitoring Support System

An automated recovery monitoring support system that includes embedded machine learning lapse prediction models powered by personal sensing could enable sustained, adaptive, and personalized monitoring. For example, it could help an individual track their risk of lapsing, alert an individual to important drivers of their lapse risk, and potentially recommend personalized recovery activities and other supports.

Moment-by-moment personal sensing of intra- and interpersonal risk factors for alcohol use disorder is already feasible [22-33]. Self-report sensing methods, like ecological momentary assessment (EMA), offer privileged access into subjective factors, such as craving, affect, valence, and self-efficacy, that may be difficult to quantify reliably through other sensing methods. More novel sensing methods, such as tracking geolocation and cellular communications, could provide a window into risk-relevant information difficult to obtain with self-report. For example, individuals may not have the insight to report on subtle changes in routine or changes to one’s social circle. Other information, such as time spent in risky locations, could not possibly be collected solely by self-report without drastically increasing the burden of EMA (eg, by increasing the number of questions or frequency of prompts). Irrespective of the method used, sensing lapse risk factors can provide ongoing monitoring that cannot be accomplished in real time by clinicians and is difficult for patients to implement consistently on their own.

Machine learning models can predict future alcohol use [28,33,34] and lapses [32,35] using these sensed features. Our group has developed a model that uses EMA, collected 4 times daily, to predict future lapses back to drinking in the next day [35]. Critically, the model’s performance exceeded general benchmarks for excellent performance [36], with an area under the receiver operating characteristic curve of 0.91, when rigorously assessed on new data from new individuals.

Methods from interpretable machine learning [37] can be used to identify patient-specific predictors of lapse risk at any moment in time [35]. These outputs can be anchored within the relapse prevention model to identify specific interventions and supports that are risk-relevant for each patient—much like a clinician would do if they were available in the moment. For example, during sensed periods of high stress, guided mindfulness and body scans could be recommended. Moreover, these systems can harness valuable information that a clinician could not realistically capture, like real-time changes in activity patterns. If increased time with risky people or locations is driving lapse risk, the recovery monitoring support system could provide information on local Alcoholics Anonymous or other support meetings.

Beyond exceptional performance and interpretable inputs, it is imperative that feedback from these machine learning models is intentionally tailored such that patients use the information and follow its recommendations. Communication research has begun to explore how humans communicate with artificial intelligence and other embedded algorithms [38,39] and how qualities of these communications influence trust, acceptance, and the use of “advice” [39-44].

The degree of transparency of the model’s outputs (ie, the system’s reasoning) is one potential area for tailoring. Research has demonstrated that making predictions or recommendations from “black-box” machine learning models more transparent can improve perceptions, acceptance, and trust of these embedded machine learning models [45-51]. In the context of a recovery monitoring support system, some transparency, such as revealing key factors driving an individual’s lapse risk, may also promote patient learning and insight to yield additional clinical benefits [46,52-54]. However, in some instances, overly complex or otherwise detailed explanations of how a system works may erode confidence in that system because incremental feedback provides more information than necessary, reveals errors, or leads users to question the system even when it was correct [45,48,51,52,55-57].

Research also suggests that people are more likely to trust, prefer, and engage with automated systems that portray human-like traits, emotions, and intentions [58-60]. Tailoring the linguistic style and tone may be one way to deliver feedback in a manner that more closely resembles an authentic supportive interaction.

Automated communications written with an informal linguistic style have been shown to be related to higher perceptions of human-likeness and trust compared to formal styles. However, this relationship between style and trust is likely context-dependent. In studies where interactions with automated agents are more task- and goal-oriented (eg, during a customer service interaction), informal style was associated with perceived human-likeness, but not trust or overall satisfaction [61,62].

Substantial research exists on human-written (rather than automated) supportive messages and advice. Messages written in tones that acknowledge the recipient’s feelings, help the recipient feel accepted, validate experiences of distress, and emphasize the feasibility of following the advice or recommendation have all been shown to be important [63-65]. Given that people tend to anthropomorphize automated messaging systems, these tones are likely important when extending to a supportive messaging system. It is less clear, however, which tones are most appropriate and would be best received in the context of a recovery monitoring support system that delivers important information about lapse risk.

Additionally, important individual differences in linguistic style and tone preferences exist. Research shows that people tend to rate automated messages and agents more favorably when they reflect traits similar to their own (ie, the similarity-attraction effect [66]). These findings have been demonstrated to exist broadly at the group level (eg, culture [67], gender [68,69], and age [70]) and narrowly at the individual level (eg, cognitive and personality style [71,72]).

In the context of digital mental health applications, more broadly, promoting trust is important for eventual therapeutic benefit [73], and mistrust is a key factor in abandonment of mobile health apps [74]. Moreover, declines in user engagement are a known issue in the context of digital mental health supports and may not be exclusively related to trust in the system [75]. Therefore, for a recovery monitoring support system to succeed, we must first explicitly evaluate and optimize the feedback from these embedded machine learning models to maximize patient engagement, trust, and clinical benefit.

### Objectives

Our broad goal is to develop a recovery monitoring support system with personalized daily support messages for people with alcohol use disorder. In this project, we propose to optimize the components included in these daily support messages to increase engagement with the support system.

Our approach is guided by the Multiphase Optimization Strategy (MOST) framework [76-78]. MOST has become highly influential for the optimization and evaluation of adaptive and multicomponent interventions in digital health. Its core assertions are that (1) interventions should be explicitly optimized to meet specific criteria; (2) intervention optimization and intervention evaluation are different phases of research, pursue different specific aims, and require different methodological approaches; and (3) the optimization of an intervention should precede its evaluation. The research in this project focuses on the MOST optimization phase. Completion of study objectives sets the stage for future, programmatic MOST research to evaluate optimized smart digital therapeutics using appropriate designs (eg, randomized controlled trial) for subsequent phases of development.

We will manipulate 4 candidate components of daily support messages that convey transparent, individualized, risk-relevant information from our machine learning lapse prediction model to participants. These message components include (1) the user’s current lapse probability for that day and trends in that daily lapse probability over the past 2 weeks, (2) an important lapse feature contributing to their current lapse probability, (3) a risk-relevant recommendation for a recovery activity to complete that day, and (4) linguistic style and tone personalization for the support message. These components use output that would be available from any machine learning lapse prediction model such that conclusions about the impact of these components on engagement can generalize beyond our system.

Models will be optimized on a measure of engagement (days of engagement with the daily support messages). We will use a MOST factorial experiment to determine which of the 4 message components encourages individuals to continue engaging with the support system. We will also look at secondary optimizing outcomes of trust in the support system, perceived usefulness of the support messages, and digital working alliance with the support system, as these are likely to drive long-term engagement.

In addition to the engagement outcome, we will test message component effects on clinical outcomes because the risk-relevant information from our machine learning model may provide direct benefits to participants through mechanisms other than engagement (eg, information about relapse processes highlighted by important model features may promote gradual adaptive lifestyle and behavioral adjustments that are difficult to quantify). Message components that either increase engagement or improve clinical outcomes will be recommended for use in future recovery monitoring support systems and digital therapeutics.

## Methods

### Overview

The primary goal of this study is to optimize daily support messages from our Smart Technology for Addiction Recovery (STAR) recovery monitoring support system to increase participant engagement with the system. To this end, participants will receive daily support messages from the STAR system for 16 weeks. We will manipulate 4 separate components of these support messages independently in a fully crossed, 4-way factorial, between-subjects design. Each component is operationalized to be binary (eg, include or exclude) so that each is either included or excluded in the support message delivered to the participant. This yields 16 between-subjects conditions, with each participant assigned to receive support messages defined by one of these conditions for the entire study period.

The first 3 components of the support messages involve personalized information based on output from the lapse risk prediction model embedded within the STAR system. The three prediction model-based components are (1) the user’s current lapse probability for that day and trends in that daily lapse probability over the past 2 weeks, (2) an important lapse feature contributing to their current lapse probability, and (3) a risk-relevant recommendation for a recovery activity to complete that day.

The fourth component of the support message is linguistic style and tone personalization. The support messages are created using a large language model (LLM) that takes the relevant prediction model-based components as inputs along with a prompt that dictates the linguistic tone and style of the message. Participants either receive support messages using a linguistic tone and style that matches their prespecified preferences or is yoked to another participant’s preferences.

The primary analyses for this study evaluate the main effects and 2-way interactions among these 4 message components on days of engagement with the STAR system daily support messages. The secondary analyses examine the effects of the components on other perceptions of the STAR system (message usefulness, system trust, and system digital working alliance) and clinical outcomes (eg, days of drinking and heavy drinking and flourishing).

### Participants

Participants must be 18 years of age or older and meet criteria for alcohol use disorder with at least moderate severity (≥4 *Diagnostic and Statistical Manual of Mental Disorders [Fifth Edition;* *DSM-5*] symptoms). All participants will report a goal of abstinence from alcohol, with their most recent use of alcohol between 1 week and 3 months in the past at study intake [79]. Participants with medical and psychiatric comorbidities will not be excluded. However, participants must be able to read in English and will be excluded if they have disabilities that prevent the use of a smartphone (eg, uncorrected vision, hearing problems, or profound cognitive impairment). Participants must have a smartphone and cellular plan.

We will recruit 304 individuals to participate for up to 17 weeks. This includes a 1-week burn-in period to collect input features prior to using the lapse risk prediction model for personalized support messages followed by 16 weeks of daily support messages from the STAR system combined with data collection of study outcomes. Participants will be recruited using social media and through laboratory-affiliated treatment centers. A diverse sample with respect to age, sex, race or ethnicity, and population density (eg, urban, suburban, and rural regions) will be recruited. We will track recruitment success and make ongoing adjustments to meet our target sample characteristics throughout the recruitment period.

Participants will be paid US $40 for the intake visit and US $20 for phone visits at 8 and 16 weeks into the data collection period. Participants will also be paid up to US $105 each month on study for completing daily EMAs (up to US $50 per month depending on adherence), for sharing geolocation data (US $5 per month), and to offset costs of cell phone service (US $50 per month).

### Procedure

Participants will first complete an intake session by phone or videoconference according to their preference. During this session, study staff will describe study procedures, requirements, and participant compensation. Study staff will also confirm the participant’s alcohol use disorder diagnosis (via module E of the Structured Clinical Interview for *DSM-5*) and assess other inclusion or exclusion criteria to determine study eligibility (eg, abstinence goal and duration). Eligible participants will be randomly assigned to 1 of 16 possible message conditions (a crossed factorial design of 4 message components included or not included in the messages).

**Figure 1:**
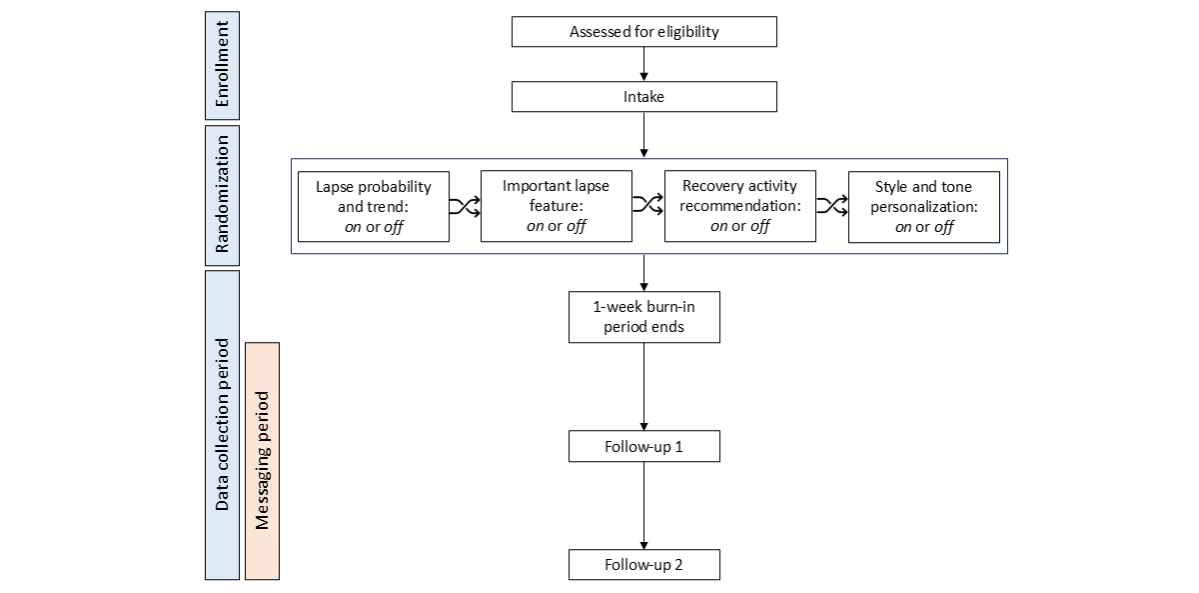
Participant flow diagram. During enrollment, participants are assessed for eligibility and complete baseline measures. Eligible participants are subsequently randomized to 1 of 16 possible messaging conditions. Each of the 4 message components, lapse probability and trend, important lapse feature, recovery activity recommendation, and style and tone personalization, can be turned on or off (ie, 2×2×2×2 factorial design). Participants then immediately begin providing ecological momentary assessment and geolocation data. After a 1-week burn-in period, participants begin receiving daily support messages. Participants complete 2 follow-up visits at 8 and 16 weeks after messaging begins. Each follow-up visit consists of an interview and a battery of self-report measures.

Following the intake session, participants will start to complete daily EMAs and provide geolocation data. After a 1-week burn-in period, participants will begin receiving personalized daily support messages based on output from the lapse risk prediction model embedded in the STAR system (see Daily Support Messages section). Participants will continue to complete EMAs, share geolocation, and receive daily support messages for 16 weeks following the burn-in period. Participants will complete 2 phone follow-up visits at 8 and 16 weeks into this data collection period to measure other study outcomes. Figure 1 presents a flow diagram of study participation.

### Lapse Risk Prediction Machine Learning Model

Following the methods and analysis workflow used in Wyant et al [35], we have developed a machine learning model that uses both geolocation and daily EMAs to predict the probability of a lapse back to alcohol use in the next day. This elastic net model was trained using approximately 300,000 labeled observations from 146 participants with moderate to severe alcohol use disorder [31,35]. Critically, the model has high combined sensitivity and specificity as indicated by its area under the receiver operating characteristic curve that exceeds 0.90 for held-out observations (using grouped k-fold cross-validation). This model can be used to provide both the probability of a lapse back to alcohol use in the next 24 hours and the change in that lapse probability over the past 2 weeks (eg, increasing, decreasing, or stable). A complete description of the model, including model coefficients and feature engineering details, is available on the Open Science Framework (OSF) repository for this project [80].

Local feature importance [37] for risk categories (eg, craving, stress, and risky situations) is quantified in log-odds using model coefficients and scores for features within each risk category that are associated with each model prediction (ie, a predicted lapse probability for a specific participant on a specific day). Locally important features are features that substantially increase or decrease the predicted probability of a lapse for that participant at that time from the aggregate lapse probability (ie, average probability across all participants and times in the original training data). We consider features that increase or decrease the log-odds of a lapse by >|0.1|, clinically important for binary classification models [37]. By extracting local (ie, for a specific, single prediction) feature importance, we can use the model to determine not only the probability of a lapse and its change over time but also information about why that lapse is likely or unlikely to happen.

Our model features tap into key constructs from the relapse prevention model such as craving, affect, stressors, lifestyle imbalances, high-risk situations, self-efficacy or confidence, and abstinence violation effects. This allows us to use the relapse prevention model to identify interventions and other supports that are personally relevant to that participant at that moment in time to address their risk. Following methods implemented by Fisher et al [81] and Fernandez et al [82] (also see [83-86]), combinations of lapse probability (categorized into low, moderate, or high risk) and important feature categories are mapped to specific recommendations using a mapping matrix based on clinical expertise guided by the relapse prevention model. Combinations of lapse probability and important features (ie, cells of the mapping matrix) often map to more than 1 appropriate module. For example, if craving is important and lapse probability is low, multiple useful urge management recommendations exist (eg, guided urge surfing and distracting games and activities). Thus, this procedure allows us to use the lapse prediction model to identify the set of interventions and supports that are most personally relevant to the participant at that moment in time, given their recent experiences, current lapse probability, and important risk features.

### Daily Support Messages

#### Overview

All participants will receive daily support messages beginning after the 1-week burn-in period. Each day, participants are sent an SMS text message that will contain a Qualtrics (Qualtrics XM) link. The daily support message is accessed and viewed or read in Qualtrics by clicking on that link. The use of Qualtrics to display the support message allows us to have a time-stamped confirmation that the support message has been accessed that day. Text messages with this Qualtrics link will be sent out at 5 AM each morning in the participant’s own time zone so that the support message is available at the start of the new day upon waking. A reminder is also sent by text message at 11 AM and 4 PM if the participant has not yet accessed the support message in Qualtrics by those times.

The support messages are generated for each participant each night using an LLM (GPT-4o) that is accessed by the STAR system through the Microsoft 365 Copilot application programming interface. LLM prompts and example messages are provided on the OSF repository for this project [80]. The core of each support message is a simple statement that encourages the participant to engage with their recovery that day. In addition to the core statement, the message may include up to 4 additional components of personalized information derived from their linguistic tone or style preferences and the lapse risk prediction model to guide their recovery efforts that day. These components are (1) their current lapse probability for that day and trends in that daily lapse probability over the past 2 weeks, (2) an important lapse feature contributing to their current lapse probability, (3) a risk-relevant recommendation for a recovery activity to complete that day, and (4) linguistic style and tone personalization. These 4 message components are operationalized as follows.

#### Current Lapse Probability and Trends in Daily Lapse Probability Over the Past 2 Weeks

If included, the support message will contain categorical information about the participant’s lapse probability in the next 24 hours based on output from the lapse risk prediction model. Lapse probability will be provided using a 3-level categorization of “low risk” (*P*≤.05), “moderate risk” (.05<*P*≤.20), or “high risk” (*P*>.20). These probability cut points were determined both from clinician input and distributional information in the sample of 151 participants with alcohol use disorder where we developed our model [35]. The cut points represent approximately the 10th and 60th percentiles in the overall distribution of the ~300,000 observed probabilities. The decision to convey lapse probability categorically (rather than numerically) follows current best practice recommendations to avoid complex numerical probability statements, especially for those with low numeracy, and provide the minimal information needed for individuals to assess the magnitude of risk, weigh options, and act [87-91]. It also follows the format from an existing app that characterizes the risk associated with various blood alcohol concentration levels [92].

If included, the support message will also contain categorical information about the trend in the predicted probabilities of a lapse over the past 2 weeks. Lapse probability change will be reported using a 3-level categorization of “decreasing risk” (lapse probability decreases by ≥.05 over 2 weeks), “increasing risk” (probability increases by ≥.05 over 2 weeks), or “stable risk” (probability changes by <.05 over 2 weeks). Lapse probability change is quantified as the difference in mean weekly predicted lapse probability over the 2 previous weeks (ie, week 2 mean–week 1 mean). We use a simple weekly mean difference to capture both linear and more complex (eg, increasing monotonic) trends across these 2 weeks. The 2-week lapse probability trend is provided because trajectory information may make subtle changes in risk level more apparent, can relieve cognitive load associated with monitoring over time, and can provide incremental value beyond absolute risk levels alone [93]. The decision to convey this information categorically follows the same best practice recommendations as for lapse probability [87-92].

#### Important Lapse Feature

If included, the support message will contain a description of 1 important model feature category that contributes to the participant’s predicted lapse probability that day. The feature category will be selected from among the set of important feature categories that day as defined based on the categories’ log-odds values (ie, feature categories that increase or decrease the log-odds of a lapse by >|0.1|). Among this set of important features, features will be ranked or sorted by how often they have been included in recent support messages and then the magnitude of their importance score. This will prioritize providing the participant with information about new but also important risk features. We include this message component because providing risk feature information may help participants both identify which issues to target for change and select appropriate support tools matched to these issues [94]. Emerging best practices from interpretable machine learning suggest that this information may make the machine learning model less of a “black box,” which may build trust, or allow individuals to engage more thoughtfully (eg, questioning false positives and scrutinizing model behavior) and gain insight [95-97].

#### Risk-Relevant Recommendation for a Recovery Activity

If included, the support message will contain a suggestion to consider completing a specific recovery activity that has been identified by the lapse risk prediction model as personally risk-relevant, given their lapse probability and important model features. All recovery activities are drawn from existing empirically based treatment protocols for relapse prevention, including matrix [98], cognitive-behavioral coping skills therapy [99], and motivational enhancement therapy [100]. As with the feature information mentioned earlier, when multiple risk-relevant recovery activities are available and appropriate, given the lapse risk prediction model output, activities that have not been recommended recently will be selected. We include this message component following best practice suggestions to pair risk information with specific action recommendations to address the risk [101]. This component follows directly from the guiding thesis of our research that improved clinical outcomes will result from increases in adaptive, personally relevant engagement rather than simply more engagement. This component also follows recent examples of digital therapeutics that used machine learning prediction models to generate intervention recommendations for medical or health issues other than alcohol use disorder [102,103].

#### Linguistic Style and Tone Personalization

In addition to the relevant lapse risk prediction model–based message components for that participant, the LLM prompt for support message creation also includes details about the linguistic style (formal or informal) and tone (legitimizing, caring and supportive, self-efficacy support, acknowledging, value affirming, and normalizing) for the support message. These styles and tones were selected based on research on linguistic factors that can affect the acceptance and use of advice during algorithmic or computer-mediated communications [44,104], medical decision-making [105-107], and communications more generally [108-111].

At intake, participants rate how much they would like to receive messages written in each of the available tones and styles independently on a 7-point Likert scale with 1=strongly disagree and 7=strongly agree as anchors. Participants are assigned to either receive support messages matched to their preferences (ie, across days, support messages will be written using any of the tones or styles that were rated higher than the neutral midpoint of the scale) or yoked to receive messages that match the preferences of another participant.

### Measures

#### Overview

Detailed descriptions of the measure items, sources, and administration are available on the project’s OSF repository [80]. Our description of study measures is organized into 5 categories: demographic and other stable characteristics, lapse prediction model inputs, primary or optimization outcome, secondary system outcomes, and secondary clinical outcomes. A summary of these measures is also presented in Table 1.

**Table 1:** Measure constructs, sources, frequency, and use.

Constructs	Source	Measurement frequency	Measure use
Geolocation	FollowMee app	Continuous	Model input
Daily experiences and events (eg, craving, affect, stressors, and risky situations)	EMA^a^	Daily	Model input
System engagement	Confirmation support message accessed	Daily	Primary or optimization outcome
Demographics	Laboratory-created	Intake visit	Baseline characterization or covariate
Alcohol use disorder symptoms	*Diagnostic and Statistical Manual of Mental Disorders (Fifth Edition)*	Intake visit	Baseline characterization or covariate
Alcohol use history	Laboratory-created	Intake visit	Baseline characterization or covariate
Lifetime substance use	World Health Organization’s Alcohol, Smoking, and Substance Involvement Screening Test	Intake visit	Baseline characterization or covariate
Trust in automated systems	Adapted from Propensity to Trust Questionnaire and Trust in Automation Scale	Intake visit	Baseline characterization or covariate
Message usefulness	Laboratory-created	8- and 16-week follow-up visits	Secondary system outcome
System trust	Adapted from Trust of Automated Systems Test and Trust in Automation Scale	8- and 16-week follow-up visits	Secondary system outcome
System digital working alliance	Digital Working Alliance Inventory	8- and 16-week follow-up visits	Secondary system outcome
Number of drinking days (past 30 days)	Alcohol Timeline Follow-Back and EMA	8- and 16-week follow-up visits	Secondary clinical outcome
Number of heavy drinking days (past 30 days)	Alcohol Timeline Follow-Back and EMA	8- and 16-week follow-up visits	Secondary clinical outcome
Anxiety	Generalized Anxiety Disorder-7	Intake and 8- and 16-week follow-up visits	Secondary clinical outcome
Depression	Patient Health Questionnaire-9	Intake and 8- and 16-week follow-up visits	Secondary clinical outcome
Human flourishing	Flourishing Measure	Intake and 8- and 16-week follow-up visits	Secondary clinical outcome
Recovery capital	Multidimensional Inventory of Recovery Capital	Intake and 8- and 16-week follow-up visits	Secondary clinical outcome

^a^EMA: ecological momentary assessment.

#### Demographic and Other Baseline Characteristics

At the intake visit, we will collect self-report information about demographics (age, sex at birth, gender identity, sexual orientation, race, ethnicity, household income, education level, marital status, and number of individuals living in the household), *DSM-5* alcohol use disorder symptoms, general alcohol use history characteristics (eg, age of first use, years of regular use, number of quit attempts, and previous treatment received), and lifetime substance use (World Health Organization’s Alcohol, Smoking and Substance Involvement Screening Test [112]). We also measure individual differences in generic trust in automated systems using a subset of items from the Propensity to Trust Questionnaire (6 items [113]) and the Trust in Automation scale (7 items [114]).

#### Lapse Prediction Model Inputs

Input features for the lapse risk prediction model are engineered from two sources: (1) 1 time daily EMA and (2) passively sensed geolocation. Participants will complete 1 brief (less than 1 minute to complete) EMA each day. EMAs will be sent to the participant by SMS text message at 5 PM each night in the participant’s own time zone. These SMS text messages will include a link to a Qualtrics survey optimized for completion on their smartphone. A reminder to complete the survey is sent by text message at 7 PM if a participant has not yet completed it.

On each EMA, participants will report their current mood (ie, affective valence and arousal), their peak alcohol craving since their last EMA, and the occurrence and intensity of any positive events and any stressors or hassles since their last EMA. They also report how likely they are to encounter risky situations (people, places, or things) and pleasant and stressful events in the next week and how confident they are about abstaining from alcohol use in the next week. They conclude each EMA by reporting any alcohol use that they have not yet reported, the time of the start of that use, the duration of the drinking episode, and the number of drinks consumed.

Participants’ location will be continuously sensed passively using the FollowMee (FollowMee LLC) geolocation tracking app. We will increase the predictive signal from geolocation by gathering contextual information about the locations that participants visit frequently (≥2 times per month). For frequent locations, participants will report the location type (eg, home of friend, bar, restaurant, workplace, and Alcoholics Anonymous meeting location), if alcohol is typically present at that location, if they drank there previously, their typical emotional experience at that location (pleasant, unpleasant, mixed, or neutral), and the perceived risk to their recovery associated with that location. This contextual information can be gathered quickly by appending these additional questions about a newly detected frequent location to their next daily EMA.

#### Primary or Optimization Outcome

Our primary outcome for this study is STAR system engagement. System engagement is measured by counting the days that participants access the daily support message by following the link to the support message provided to them by text message. We will calculate message engagement scores that count the number of days the support message is accessed across the full 16 weeks of data collection. Support message engagement serves as the optimization outcome for this study.

#### Secondary System Outcomes

We measure 3 secondary STAR system outcomes at 8 and 16 weeks into the data collection period: daily support message usefulness, system trust, and system digital working alliance. Daily support message usefulness is measured with 7 items scored on a 7-point Likert scale. These items ask participants to rate the perceived helpfulness, general liking, and personal relevance of the messages [115-118]. Trust in the overall STAR recovery monitoring support system is measured with 10 items scored on a 7-point Likert scale. These items come from the Trust of Automated Systems Test (9 items [119]) and the Trust in Automation scale (1 item [114]). The system digital working alliance between the participant and the STAR system is measured with the Digital Working Alliance Inventory [120]. This measure consists of 6 items measured on a 7-point Likert scale.

#### Secondary Clinical Outcomes

We will measure 2 primary clinical outcomes recommended by the Food and Drug Administration [121] for the evaluation of interventions for alcohol use disorder: number of drinking days and number of heavy drinking days in the past 30 days. These 2 outcomes are quantified at 8 and 16 weeks into the data collection period. Information about participant alcohol use is obtained from 2 sources. First, participants report episodes of alcohol use in the daily EMAs as described earlier. Second, study staff conduct a 30-day alcohol Timeline Follow-Back (TLFB) procedure [122] at the study phone visits at 8 and 16 weeks in the data collection period. The TLFB procedure is a calendar-assisted retrospective reconstruction of how many standard alcoholic drinks were consumed by the participant each day in the assessment period. This procedure is further enhanced by adding previously reported drinking episodes (dates, times, and number of drinks) from the daily EMAs to the calendar used with the participant. This provides an opportunity to validate those previous reports and collect any additional drinking episodes that may have been missed by the EMAs. The number of drinking days is defined as the number of days that any alcohol is consumed during the relevant 30-day period. The number of heavy drinking days is defined as the number of days that more than 3 or 4 standard drinks are consumed for women and men, respectively, during the relevant 30-day period. The TLFB will also be administered by study staff during the intake visit to determine inclusion or exclusion criteria (ie, time since last drink).

We will also collect secondary measures of anxiety (General Anxiety Disorder-7 [123]), depression (Patient Health Questionnaire-9 [124]), human flourishing (total score and subscales from the Flourish Measure [125]), and recovery capital (Multidimensional Inventory of Recovery Capital [126]). Each of these measures will be collected at intake and at 8 and 16 weeks into the data collection period.

### Data Analytic Plan

#### Overview

All data preprocessing, visualization, and exploratory analyses will be done using the tidyverse ecosystem [127] in R (R Foundation for Statistical Computing). General or generalized mixed effects models will be fit using the *lme4* package [128] in R. Multiple imputation will be performed using the *mice* package [129].

#### Primary Analyses

MOST [77] highlights the importance of optimizing any intervention prior to evaluating it with a formal randomized controlled trial. The goal of this project is to optimize support message components in the STAR system by identifying which of the several message components increase engagement with the daily support messages. Such information will be crucial to develop future digital therapeutics for alcohol and other substance use disorders that use personalized supportive messages based on machine learning lapse risk prediction models.

Collins [130] advocates for the use of the factorial experiment as a highly efficient and powerful design to optimize intervention components generally. We use this design to evaluate the effects of our 4 message components (current lapse probability and trends in daily lapse probability over the past 2 weeks, important feature contributing to current lapse probability, risk-relevant recommendation for a recovery activity, and linguistic style and tone personalization) on the primary or optimization outcome (count of days of support message engagement) measured at the end of the data collection period (week 16). The goal of these analyses is to determine the effect of each support message component and whether the effect of one component varies depending on the level or setting of another component (eg, Is the effect of a recovery activity recommendation greater if given in combination with lapse probability or an important feature?).

We will analyze support message engagement in a generalized linear model (with Poisson or negative binomial distribution dependent on the distribution diagnostics for the count data). We will use unit-weighted orthogonal contrasts to code for main effects and 2-way interactions among the 4 message components. Support message engagement will be indexed as the count of days on which the support message was accessed by the participant across the full 16 weeks in the data collection period. Baseline covariates will be included in the model, as described in the Covariates section. This analysis will include all participants who were randomized into the study, following intention-to-treat principles. If participants discontinue use of the STAR system during the data collection period, they will get a 0 for each subsequent day they do not engage with the message.

#### Secondary Analyses

Analyses for secondary outcomes will follow the same analytic plan as for the primary analyses but will use the secondary outcomes (eg, daily support message usefulness, system trust, system digital working alliance, and clinical outcomes) as the outcome variable. These analyses will allow us to determine whether the effects of message components on engagement also extend to other perceptions of the STAR system and clinical outcomes. The error distribution (Gaussian, Poisson, and negative binomial) for these generalized linear models will be selected based on the distribution of the outcome variable. These secondary analyses will use mean scores for each outcome across the 16 weeks (ie, average of scores at 8 and 16 weeks) as the dependent measure because the effects of time are not a primary focus, given the relatively short duration of the study. These analyses will only include participants who provided at least 1 measurement for the secondary outcomes during the data collection period (ie, at 8 or 16 weeks). For participants missing 1 measurement, scores will be imputed using multiple imputation (see Missing Data section).

#### Covariates

The use of covariates has been demonstrated to improve estimation efficiency and increase power to test parameter estimates in linear models [131-135]. Given this, the inclusion of baseline characteristics measured prior to assignment to levels for message components will be considered as covariates in the primary and secondary analyses. When available, the baseline scores associated with the study outcome for each model (eg, baseline flourishing for the model examining flourishing) will be included in the model to control for baseline differences on that measure. In addition, following well-established practices in our laboratory [136-138], other baseline characteristics (eg, demographics and alcohol use or history measures and baseline scores on other outcomes) will be included in the model if they demonstrate a significant relationship with the outcome variable for that model, independent of (ie, controlling for) message component effects. This allows for the selection of covariates that will increase statistical power but not bias the parameter estimates for the message component effects.

#### Sample Size Planning

The primary optimization outcome (count of days of support message engagement) will be analyzed in a generalized linear model (with Poisson or negative binomial distribution) and contrast coding for the main effects and 2-way interactions of the 4 message components. We planned the sample size for this study by simulating the power to detect main effects or interactions of the message components across varying effect sizes and baseline (all message components off) support message engagement rates. We simulated count data using the Poisson distribution for support message engagement across 112 days (16 weeks), with λ (mean count for distribution) calculated as the number of days of observation multiplied by the baseline engagement rate. We added a message component effect to these data by increasing the count to reflect an increased rate of support message engagement across days (eg, on 5% more days) when the component was included in the support message. These simulations indicated that 304 participants would provide 85% power to detect a message component main effect or interaction that increased support message engagement rates by 3% of days when the baseline engagement rate was 85% of days. Power was higher still when message effects were larger (ie, >3%) or the baseline engagement rate was lower (eg, <85% of days). We believe that message components that increase engagement rates by less than 3% would not be sufficiently large to warrant including that component in future versions of the STAR system. Furthermore, if baseline engagement exceeds 85%, there would not be much need for support messages to increase engagement further. Therefore, we believed these bounds to be appropriate to represent the minimal clinically meaningful contribution from message components. It should also be noted that this power estimate is likely conservative because it does not include potential covariates that will be included in the analysis model.

#### Missing Data

We pursue a variety of methods to minimize biases that can occur when participants are dropped from analyses because of discontinued participation or missing data. First, we follow guidelines and procedures that have been recommended for clinical trials [139,140].

Second, our primary optimization outcome (support message engagement) will use all participants who were randomized to the study because days of support message engagement can be measured for all participants who were randomized, even if they do not complete the full 16 weeks of data collection.

Third, our Food and Drug Administration–recommended clinical outcomes (days of drinking and heavy drinking) are somewhat robust to missing data because they can be scored independently from 2 separate methods (daily EMA and TLFB at 8- and 16-week visits). Ideally, both measurement methods will contribute to the most reliable measurement of these outcomes. However, either is sufficient to score these clinical outcomes and can be used in isolation if the other method is missing (due to missing periods for daily EMA or missing study visits).

Finally, our secondary analyses use outcomes that are averaged across 2 measurement periods. This will provide a more reliable measurement for the outcomes. In instances where participants are missing a measurement, we will use multiple imputation to estimate the missing score. Multiple imputation does not attempt to estimate each missing value with a single imputed value but instead uses a random sample of imputed values. This allows for valid statistical inferences that properly reflect the uncertainty that results from missing values, such as valid SEs and CIs for parameters [141]. In brief, we will use *mice* to impute missing values in the dataset using predictive mean matching. Five distinct imputed datasets will be generated. Relevant statistical models for each secondary analysis will be fit to these imputed datasets, and parameter estimates will be averaged.

### Ethical Considerations

The institutional research board of the University of Wisconsin-Madison Health Sciences approved this project (IRB #2024-0869). All participants will provide written informed consent and will be told they can discontinue at any time without penalty. Participants will share location data through the use of a third-party app (FollowMee). Their location data will only be identified by a randomly generated Device ID created when the app is installed. Location data are encrypted and sent to the FollowMee servers, where it will be automatically deleted after 14 days. The University of Wisconsin Cybersecurity Office has reviewed the FollowMee software, its security practices, data loss history, and encryption system for transmitting data and judged the risk of a data breach to be low. All raw data (including geolocation) will be stored on Health Insurance Portability and Accountability Act (HIPAA)–compliant servers protected behind a firewall. Raw data will be labeled with an anonymous study identification number. We also have obtained a certificate of confidentiality, which prevents the disclosure of identifiable, sensitive research information to anyone not connected to the study except with explicit consent by the participant. While we use an LLM to generate personalized support messages, no identifiable participant data will be passed into the LLM. It will only be given up to 4 pieces of information about the participant: lapse probability (high, moderate, or low), trend in lapse probability (increasing, decreasing, or stable), one important lapse feature category selected from several categories, and preferences for style and tone.

## Results

The National Institute on Alcohol Abuse and Alcoholism funded this project (R01AA031762) on August 9, 2024, with a funding period from August 20, 2024, to July 31, 2029. Enrollment will begin in December 2025. We plan to recruit participants for approximately 3 years.

## Discussion

Clinical observation and research suggest that successful recovery from alcohol use disorder requires long-term and perhaps lifelong self-monitoring of lapse risk. Self-monitoring, however, is difficult given the dynamic and complex interplay of numerous risk factors over time.

Machine learning lapse prediction models are now emerging. Recovery monitoring support systems that synthesize these developments can potentially guide patients toward personally risk-relevant engagement that is adaptive, efficient, and more effective. Research is needed to determine the optimal feedback from embedded machine learning prediction models such that patients make the best use of these automated capabilities.

The optimization study in this project is guided by the MOST framework. The research in this project focuses on the MOST optimization phase. We propose to optimize feedback from a lapse prediction model (via daily engagement messages) both to increase engagement with a recovery monitoring support system and to improve clinical outcomes. We will evaluate the effects of 4 message components (lapse probability or lapse probability change, an important feature, a risk-relevant module recommendation, and tone personalization) that comprise the daily engagement messages and can make the output from our machine learning model more transparent to participants. Completion of our specific aims sets the stage for future, programmatic MOST research to evaluate optimized recovery monitoring support systems using appropriate designs (eg, randomized controlled trial) for that subsequent phase of development.

Additionally, the optimization components use output that would be available from any machine learning lapse prediction model such that conclusions about the impact of these components on engagement can generalize beyond our specific machine learning model. Similarly, engagement messages including these message components could be used in any recovery monitoring system or digital therapeutic for alcohol use disorder, allowing conclusions to generalize to current and future system variants. At the conclusion of the grant period, we will also deliver this optimized recovery monitoring support system as a tangible product and model for how to embed sensing and machine learning into other existing systems.

## Appendix

Multimedia Appendix 1 Peer review report by the National Institute on Alcohol Abuse and Alcoholism (R01AA031762).

## Abbreviations

DSM-5: Diagnostic and Statistical Manual of Mental Disorders (Fifth Edition)
EMA: ecological momentary assessment
HIPAA: Health Insurance Portability and Accountability Act
LLM: large language model
MOST: Multiphase Optimization Strategy
OSF: Open Science Framework
STAR: Smart Technology for Addiction Recovery
TLFB: Timeline Follow-Back

## Data Availability

The datasets generated or analyzed during this study will be available in the Open Science Framework repository upon completion of the study [80].

## References

[1] 2021 National Survey on Drug Use and Health (NSDUH-2021-DS0001). SAMHSA Center for Behavioral Health Statistics and Quality.

[2] Centers for Disease ControlPrevention (CDC) Annual average for United States 2011-2015 alcohol-attributable deaths due to excessive alcohol use, all ages. 2022 Alcohol Related Disease Impact (ARDI) Application Website.

[3] Esser MB, Leung G, Sherk A, Bohm MK, Liu Y, Lu H, Naimi TS (2022). Estimated deaths attributable to excessive alcohol use among US adults aged 20 to 64 years, 2015 to 2019. JAMA Netw Open.

[4] Substance Abuse and Mental Health Services Administration (US), Office of the Surgeon General (US) (2016). Facing Addiction in America.

[5] Dennis M, Scott CK (2007). Managing addiction as a chronic condition. Addict Sci Clin Pract.

[6] McLellan AT, Lewis DC, O'Brien CP, Kleber HD (2000). Drug dependence, a chronic medical illness: implications for treatment, insurance, and outcomes evaluation. JAMA.

[7] Marlatt GA, Gordon JR (1985). Relapse Prevention: Maintenance Strategies in the Treatment of Addictive Behaviors.

[8] Marlatt GA, Donovan DM (2007). Relapse Prevention, Second Edition: Maintenance Strategies in the Treatment of Addictive Behaviors.

[9] McHugh RK, Hearon BA, Otto MW (2010). Cognitive behavioral therapy for substance use disorders. Psychiatr Clin North Am.

[10] Liese BS, Beck AT (2022). Cognitive-Behavioral Therapy of Addictive Disorders. 1st Edition.

[11] Bowen S, Chawla N, Grow J, Marlatt GA (2021). Mindfulness-Based Relapse Prevention for Addictive Behaviors: A Clinician's Guide.

[12] Yaghubi M, Zargar F (2018). Effectiveness of mindfulness-based relapse prevention on quality of life and craving in methadone-treated patients: a randomized clinical trial. Addict Health.

[13] Ramadas E, Lima MPD, Caetano T, Lopes J, Dixe MDA (2021). Effectiveness of mindfulness-based relapse prevention in individuals with substance use disorders: a systematic review. Behav Sci (Basel).

[14] Goldberg SB, Tucker RP, Greene PA, Davidson RJ, Wampold BE, Kearney DJ, Simpson TL (2018). Mindfulness-based interventions for psychiatric disorders: a systematic review and meta-analysis. Clin Psychol Rev.

[15] Hendershot CS, Witkiewitz K, George WH, Marlatt GA (2011). Relapse prevention for addictive behaviors. Subst Abuse Treat Prev Policy.

[16] Witkiewitz K, Marlatt GA (2004). Relapse prevention for alcohol and drug problems: that was Zen, this is Tao. Am Psychol.

[17] Hufford MR, Witkiewitz K, Shields AL, Kodya S, Caruso JC (2003). Relapse as a nonlinear dynamic system: application to patients with alcohol use disorders. J Abnorm Psychol.

[18] Witkiewitz K, van der Maas HLJ, Hufford MR, Marlatt GA (2007). Nonnormality and divergence in posttreatment alcohol use: reexamining the project MATCH data "another way.". J Abnorm Psychol.

[19] Witkiewitz K, Marlatt GA (2007). Modeling the complexity of post-treatment drinking: it's a rocky road to relapse. Clin Psychol Rev.

[20] Brandon TH, Vidrine JI, Litvin EB (2007). Relapse and relapse prevention. Annu Rev Clin Psychol.

[21] Witkiewitz KA, Marlatt GA (2007). Therapist's Guide to Evidence-Based Relapse Prevention. 1st Edition.

[22] Epstein DH, Tyburski M, Kowalczyk WJ, Burgess-Hull AJ, Phillips KA, Curtis BL, Preston KL (2020). Prediction of stress and drug craving ninety minutes in the future with passively collected GPS data. NPJ Digit Med.

[23] Suchting R, Hébert ET, Ma P, Kendzor DE, Businelle MS (2019). Using elastic net penalized cox proportional hazards regression to identify predictors of imminent smoking lapse. Nicotine Tob Res.

[24] Hébert ET, Suchting R, Ra CK, Alexander AC, Kendzor DE, Vidrine DJ, Businelle MS (2021). Predicting the first smoking lapse during a quit attempt: a machine learning approach. Drug Alcohol Depend.

[25] Engelhard M, Xu H, Carin L, Oliver JA, Hallyburton M, McClernon FJ (2018). Predicting smoking events with a time-varying semi-parametric Hawkes process model. Proc Mach Learn Res.

[26] Mohr DC, Zhang M, Schueller SM (2017). Personal sensing: understanding mental health using ubiquitous sensors and machine learning. Annu Rev Clin Psychol.

[27] Businelle MS, Ma P, Kendzor DE, Frank SG, Wetter DW, Vidrine DJ (2016). Using intensive longitudinal data collected via mobile phone to detect imminent lapse in smokers undergoing a scheduled quit attempt. J Med Internet Res.

[28] Soyster PD, Ashlock L, Fisher AJ (2022). Pooled and person-specific machine learning models for predicting future alcohol consumption, craving, and wanting to drink: a demonstration of parallel utility. Psychol Addict Behav.

[29] Hébert ET, Stevens EM, Frank SG, Kendzor DE, Wetter DW, Zvolensky MJ, Buckner JD, Businelle MS (2018). An ecological momentary intervention for smoking cessation: the associations of just-in-time, tailored messages with lapse risk factors. Addict Behav.

[30] Moshontz H, Colmenares AJ, Fronk GE, Sant'Ana SJ, Wyant K, Wanta SE, Maus A, Gustafson DH, Shah D, Curtin JJ (2021). Prospective prediction of lapses in opioid use disorder: protocol for a personal sensing study. JMIR Res Protoc.

[31] Wyant K, Moshontz H, Ward SB, Fronk GE, Curtin JJ (2023). Acceptability of personal sensing among people with alcohol use disorder: observational study. JMIR Mhealth Uhealth.

[32] Chih MY, Patton T, McTavish FM, Isham AJ, Judkins-Fisher CL, Atwood AK, Gustafson DH (2014). Predictive modeling of addiction lapses in a mobile health application. J Subst Abuse Treat.

[33] Bae S, Chung T, Ferreira D, Dey AK, Suffoletto B (2018). Mobile phone sensors and supervised machine learning to identify alcohol use events in young adults: implications for just-in-time adaptive interventions. Addict Behav.

[34] Walters ST, Businelle MS, Suchting R, Li X, Hébert ET, Mun EY (2021). Using machine learning to identify predictors of imminent drinking and create tailored messages for at-risk drinkers experiencing homelessness. J Subst Abuse Treat.

[35] Wyant K, Sant'Ana SJK, Fronk GE, Curtin JJ (2024). Machine learning models for temporally precise lapse prediction in alcohol use disorder. J Psychopathol Clin Sci.

[36] Hosmer Jr DW, Lemeshow S (1989). Applied Logistic Regression. 1st Edition.

[37] Molnar C (2022). Interpretable Machine Learning: A Guide For Making Black Box Models Explainable.

[38] Fortunati L, Edwards A (2020). Opening space for theoretical, methodological, and empirical issues in human-machine communication. Hum-Mach Commun.

[39] Banks J, de Graaf M (2020). Toward an agent-agnostic transmission model: synthesizing anthropocentric and technocentric paradigms in communication. Hum-Mach Commun.

[40] Hoff KA, Bashir M (2015). Trust in automation: integrating empirical evidence on factors that influence trust. Hum Factors.

[41] Lutz C, Tamó-Larrieux A (2020). The robot privacy paradox: understanding how privacy concerns shape intentions to use social robots. Hum-Mach Commun.

[42] Ibrahim MA, Assaad Z, Williams E (2022). Trust and communication in human-machine teaming. Front Phys.

[43] Guzman A (2020). Ontological boundaries between humans and computers and the implications for human-machine communication. Hum-Mach Commun.

[44] Prahl A, Van Swol L (2021). Out with the humans, in with the machines?: Investigating the behavioral and psychological effects of replacing human advisors with a machine. Hum-Mach Commun.

[45] Kizilcec RF (2016). How much information?: Effects of transparency on trust in an algorithmic interface.

[46] Kulesza T, Burnett M, Wong WK, Stumpf S (2015). Principles of explanatory debugging to personalize interactive machine learning.

[47] Lim BY, Dey AK, Avrahami D (2009). Why and why not explanations improve the intelligibility of context-aware intelligent systems.

[48] Burke M, Amento B, Isenhour P (2006). Error correction of voicemail transcripts in SCANMail.

[49] Yang R, Newman MW (2013). Learning from a learning thermostat: lessons for intelligent systems for the home.

[50] Lim B, Dey A (2009). Assessing demand for intelligibility in context-aware applications.

[51] Lim BY, Dey AK (2011). Investigating intelligibility for uncertain context-aware applications.

[52] Springer A, Whittaker S (2020). Progressive disclosure: when, why, and how do users want algorithmic transparency information?. ACM Trans Interact Intell Syst.

[53] Kulesza T, Wong WK, Stumpf S, Perona S, White R, Burnett Mm, Oberst I, Ko AJ (2009). Fixing the program my computer learned: barriers for end users, challenges for the machine.

[54] Ribeiro MT, Singh S, Guestrin C (2016). "Why should i trust you": explaining the predictions of any classifier.

[55] Abdul A, Vermeulen J, Wang D, Lim B, Kankanhalli M (2018). Trends and trajectories for explainable, accountable and intelligible systems: an HCI research agenda.

[56] Bunt A, Lount M, Lauzon C (2012). Are explanations always important? A study of deployed, low-cost intelligent interactive systems.

[57] Wickens CD, Helton WS, Hollands JG, Banbury S (2022). Engineering Psychology and Human Performance. 5th Edition.

[58] Reeves B, Nass CI (1996). The Media Equation: How People treat Computers, Television, and New Media Like Real People and Places.

[59] Nass C, Moon Y (2002). Machines and mindlessness: social responses to computers. J Soc Issues.

[60] de Visser EJ, Monfort SS, McKendrick R, Smith MAB, McKnight PE, Krueger F, Parasuraman R (2016). Almost human: anthropomorphism increases trust resilience in cognitive agents. J Exp Psychol Appl.

[61] Liebrecht C, Sander L, van Hooijdonk C (2020). Too informal? How a chatbot's communication style affects brand attitude and quality of interaction.

[62] Araujo T (2018). Living up to the chatbot hype: the influence of anthropomorphic design cues and communicative agency framing on conversational agent and company perceptions. Comput Hum Behav.

[63] Burleson BR, Samter W (2009). Consistencies in theoretical and naive evaluations of comforting messages. Commun Monogr.

[64] MacGeorge EL, Guntzviller LM, Hanasono LK, Feng B (2013). Testing advice response theory in interactions with friends. Commun Res.

[65] MacGeorge EL, Foley KA, Firgens EPC, Vanderbilt RR, Worthington AK, Hackman NM (2020). “Watchful Waiting” advice for pediatric ear infections. J Lang Soc Psychol.

[66] Montoya RM, Horton RS (2012). A meta-analytic investigation of the processes underlying the similarity-attraction effect. J Soc Pers Relat.

[67] Kim Y, Baek TH, Yoon S, Oh S, Choi YK (2017). Assertive environmental advertising and reactance: differences between South Koreans and Americans. J Advert.

[68] Eyssel F, Kuchenbrandt D, Bobinger S, de Ruiter L, Hegel F (2012). If you sound like me, you must be more human?: On the interplay of robot and user features on human-robot acceptance and anthropomorphism.

[69] Lee EJ, Nass C, Brave S (2000). Can computer-generated speech have gender? An experimental test of gender stereotype.

[70] Edwards C, Edwards A, Stoll B, Lin X, Massey N (2019). Evaluations of an artificial intelligence instructor's voice: social identity theory in human-robot interactions. Comput Hum Behav.

[71] Moon Y, Nass C (1996). How “real” are computer personalities? Psychological responses to personality types in human-computer interaction. Commun Res.

[72] Nass C, Lee KM (2001). Does computer-synthesized speech manifest personality? Experimental tests of recognition, similarity-attraction, and consistency-attraction. J Exp Psychol.

[73] Torous J, Roberts LW (2017). Needed innovation in digital health and smartphone applications for mental health: transparency and trust. JAMA Psychiatry.

[74] Murnane EL, Huffaker D, Kossinets G (2015). Mobile health apps: adoption, adherence, and abandonment.

[75] Torous J, Nicholas J, Larsen ME, Firth J, Christensen H (2018). Clinical review of user engagement with mental health smartphone apps: evidence, theory and improvements. Evid Based Ment Health.

[76] Collins LM, Murphy SA, Strecher V (2007). The Multiphase Optimization Strategy (MOST) and the sequential multiple assignment randomized trial (SMART): new methods for more potent eHealth interventions. Am J Prev Med.

[77] Collins LM (2018). Optimization of Behavioral, Biobehavioral, and Biomedical Interventions: The Multiphase Optimization Strategy (MOST).

[78] Collins LM, Kugler KC (2018). Optimization of Behavioral, Biobehavioral, and Biomedical Interventions: Advanced Topics.

[79] Hagman BT, Falk D, Litten R, Koob GF (2022). Defining recovery from alcohol use disorder: development of an NIAAA research definition. Am J Psychiatry.

[80] Wyant K, Sant'Ana S, Punturieri C, Yu J, Fronk G, Maggard C, Janssen C, Wanta S, Kornfield R, van Swol L, Curtin J (2025). Maximizing engagement, trust, and clinical benefit of AI-generated recovery support messages for alcohol use disorder: protocol for an optimization study. Open Science Framework Repository.

[81] Fisher AJ, Bosley HG, Fernandez KC, Reeves JW, Soyster PD, Diamond AE, Barkin J (2019). Open trial of a personalized modular treatment for mood and anxiety. Behav Res Ther.

[82] Fernandez KC, Fisher AJ, Chi C (2017). Development and initial implementation of the Dynamic Assessment Treatment Algorithm (DATA). PLoS One.

[83] Shah RV, Grennan G, Zafar-Khan M, Alim F, Dey S, Ramanathan D, Mishra J (2021). Personalized machine learning of depressed mood using wearables. Transl Psychiatry.

[84] Cohen ZD, DeRubeis RJ (2018). Treatment selection in depression. Annu Rev Clin Psychol.

[85] DeRubeis RJ, Cohen ZD, Forand NR, Fournier JC, Gelfand LA, Lorenzo-Luaces L (2014). The Personalized Advantage Index: translating research on prediction into individualized treatment recommendations. A demonstration. PLoS One.

[86] Hall-Flavin DK, Winner JG, Allen JD, Jordan JJ, Nesheim RS, Snyder KA, Drews MS, Eisterhold LL, Biernacka JM, Mrazek DA (2012). Using a pharmacogenomic algorithm to guide the treatment of depression. Transl Psychiatry.

[87] Zikmund-Fisher BJ (2013). The right tool is what they need, not what we have: a taxonomy of appropriate levels of precision in patient risk communication. Med Care Res Rev.

[88] Sanneman L, Shah JA (2020). A situation awareness-based framework for design and evaluation of explainable AI.

[89] Fagerlin A, Ubel PA, Smith DM, Zikmund-Fisher BJ (2007). Making numbers matter: present and future research in risk communication. Am J Health Behav.

[90] Zipkin DA, Umscheid CA, Keating NL, Allen E, Aung K, Beyth R, Kaatz S, Mann DM, Sussman JB, Korenstein D, Schardt C, Nagi A, Sloane R, Feldstein DA (2014). Evidence-based risk communication: a systematic review. Ann Intern Med.

[91] Garcia-Retamero R, Cokely ET (2013). Communicating health risks with visual aids. Curr Dir Psychol Sci.

[92] Gajecki M, Berman AH, Sinadinovic K, Rosendahl I, Andersson C (2014). Mobile phone brief intervention applications for risky alcohol use among university students: a randomized controlled study. Addict Sci Clin Pract.

[93] Eini-Porat B, Amir O, Eytan D, Shalit U (2022). Tell me something interesting: clinical utility of machine learning prediction models in the ICU. J Biomed Inform.

[94] Huckvale K, Venkatesh S, Christensen H (2019). Toward clinical digital phenotyping: a timely opportunity to consider purpose, quality, and safety. NPJ Digit Med.

[95] Lauritsen SM, Kristensen M, Olsen MV, Larsen MS, Lauritsen KM, Jørgensen MJ, Lange J, Thiesson B (2020). Explainable artificial intelligence model to predict acute critical illness from electronic health records. Nat Commun.

[96] da Cruz HF, Pfahringer B, Martensen T, Schneider F, Meyer A, Böttinger E, Schapranow M (2021). Using interpretability approaches to update "black-box" clinical prediction models: an external validation study in nephrology. Artif Intell Med.

[97] Shin D (2021). The effects of explainability and causability on perception, trust, and acceptance: implications for explainable AI. Int J Hum-Comput Studies.

[98] Center for Substance Abuse Treatment (2006). Counselor's Treatment Manual: Matrix Intensive Outpatient Treatment for People With Stimulant Use Disorders. HHS Publication No. (SMA) 13-4152.

[99] DeMarce JM, Gnys M, Raffa SD, Karlin BE (2023). Cognitive Behavioral Therapy for Substance Use Disorders Among Veterans: Therapist Manual (Rev ed.).

[100] Miller WR, Zweben A, DiClemente CC, Rychtarik R, Mattson M (1999). Motivational Enhancement Therapy Manual: A Clinical Research Guide for Therapists Treating Individuals With Alcohol Abuse and Dependence.

[101] Maloney EK, Lapinski MK, Witte K (2011). Fear appeals and persuasion: a review and update of the extended parallel process model. Soc Person Psychol.

[102] Mitchell EG, Heitkemper EM, Burgermaster M, Levine ME, Miao Y, Hwang ML, Desai PM, Cassells A, Tobin JN, Tabak EG, Albers DJ, Smaldone AM, Mamykina L (2021). From reflection to action: combining machine learning with expert knowledge for nutrition goal recommendations.

[103] Goldstein SP, Evans BC, Flack D, Juarascio A, Manasse S, Zhang F, Forman EM (2017). Return of the JITAI: applying a just-in-time adaptive intervention framework to the development of m-Health solutions for addictive behaviors. Int J Behav Med.

[104] Prahl A, Van Swol L (2017). Understanding algorithm aversion: when is advice from automation discounted?. J Forecast.

[105] Dexter F, Van Swol LM (2016). Influence of data and formulas on trust in information from journal articles in an operating room management course. A & A Case Rep.

[106] Dexter F, Epstein RH, Fahy BG, Van Swol LM (2017). With directed study before a 4-day operating room management course, trust in the content did not change progressively during the classroom time. J Clin Anesth.

[107] Ahn PH, Dexter F, Fahy BG, Van Swol LM (2020). Demonstrability of analytics solutions and shared knowledge of statistics and operating room management improves expected performance of small teams in correctly solving problems and making good decisions. Perioper Care Oper Room Manag.

[108] Sniezek JA, Van Swol LM (2001). Trust, confidence, and expertise in a judge-advisor system. Organ Behav Hum Decis Process.

[109] van Swol LM, Sniezek JA (2011). Factors affecting the acceptance of expert advice. Br J Soc Psychol.

[110] Van SL, Paik JE, Prahl A (2018). Advice Recipients: The Psychology of Advice Utilization.

[111] MacGeorge EL, Swol LMV (2018). The Oxford Handbook of Advice.

[112] WHO ASSIST Working Group (2002). The Alcohol, Smoking and Substance Involvement Screening Test (ASSIST): development, reliability and feasibility. Addiction.

[113] Merritt SM, Heimbaugh H, LaChapell J, Lee D (2013). I trust it, but I don't know why: effects of implicit attitudes toward automation on trust in an automated system. Hum Factors.

[114] Körber M (2019). Theoretical considerations and development of a questionnaire to measure trust in automation.

[115] Henson P, Wisniewski H, Hollis C, Keshavan M, Torous J (2019). Digital mental health apps and the therapeutic alliance: initial review. BJPsych Open.

[116] Jensen JD, King AJ, Carcioppolo N, Davis L (2012). Why are tailored messages more effective? A multiple mediation analysis of a breast cancer screening intervention. J Commun.

[117] Tielman ML, Neerincx MA, Brinkman WP (2019). Design and evaluation of personalized motivational messages by a virtual agent that assists in post-traumatic stress disorder therapy. J Med Internet Res.

[118] Kocielnik R, Hsieh G (2017). Send me a different message: utilizing cognitive space to create engaging message triggers.

[119] Wojton HM, Porter D, T Lane S, Bieber C, Madhavan P (2020). Initial validation of the trust of automated systems test (TOAST). J Soc Psychol.

[120] Hatcher RL, Gillaspy JA (2006). Development and validation of a revised short version of the Working Alliance Inventory. Psychother Res.

[121] (2015). Alcoholism: developing drugs for treatment (No FDA D-0152-001). Food and Drug Administration.

[122] Sobell LC, Sobell MB (1992). Timeline followback: a technique for assessing self-reported alcohol consumption. Measuring alcohol Consumption: Psychosocial and Biological Methods.

[123] Spitzer RL, Kroenke K, Williams JBW, Löwe B (2006). A brief measure for assessing generalized anxiety disorder: the GAD-7. Arch Intern Med.

[124] Kroenke K, Spitzer RL, Williams JBW (2001). The PHQ-9: validity of a brief depression severity measure. J Gen Intern Med.

[125] VanderWeele TJ (2017). On the promotion of human flourishing. Proc Natl Acad Sci USA.

[126] Bowen E, Irish A, Wilding G, LaBarre C, Capozziello N, Nochajski T, Granfield R, Kaskutas LA (2023). Development and psychometric properties of the Multidimensional Inventory of Recovery Capital (MIRC). Drug Alcohol Depend.

[127] Wickham H, Averick M, Bryan J, Chang W, McGowan L, François R, Grolemund G, Hayes A, Henry L, Hester J, Kuhn M, Pedersen T, Miller E, Bache S, Müller K, Ooms J, Robinson D, Seidel D, Spinu V, Takahashi K, Vaughan D, Wilke C, Woo K, Yutani H (2019). Welcome to the tidyverse. J Open Source Softw.

[128] Bates D, Mächler M, Bolker B, Walker S (2015). Fitting linear mixed-effects models using lme4. J Stat Soft.

[129] van Buuren S, Groothuis-Oudshoorn K (2011). Mice: Multivariate imputation by chained equations in R. J Stat Soft.

[130] Collins LM (2018). Introduction to the factorial optimization trial. Optimization of Behavioral, Biobehavioral, and Biomedical Interventions: The Multiphase Optimization Strategy (MOST).

[131] Koch GG, Tangen CM, Jung JW, Amara IA (1998). Issues for covariance analysis of dichotomous and ordered categorical data from randomized clinical trials and non-parametric strategies for addressing them. Stat Med.

[132] Moore KL, van der Laan MJ (2009). Covariate adjustment in randomized trials with binary outcomes: targeted maximum likelihood estimation. Stat Med.

[133] Pocock SJ, Assmann SE, Enos LE, Kasten LE (2002). Subgroup analysis, covariate adjustment and baseline comparisons in clinical trial reporting: current practice and problems. Stat Med.

[134] Tsiatis AA, Davidian M, Zhang M, Lu X (2008). Covariate adjustment for two-sample treatment comparisons in randomized clinical trials: a principled yet flexible approach. Stat Med.

[135] Zhang M, Tsiatis AA, Davidian M (2008). Improving efficiency of inferences in randomized clinical trials using auxiliary covariates. Biometrics.

[136] Kaye JT, Fronk GE, Zgierska AE, Cruz MR, Rabago D, Curtin JJ (2019). Acute prazosin administration does not reduce stressor reactivity in healthy adults. Psychopharmacology (Berl).

[137] Moberg CA, Bradford DE, Kaye JT, Curtin JJ (2017). Increased startle potentiation to unpredictable stressors in alcohol dependence: possible stress neuroadaptation in humans. J Abnorm Psychol.

[138] Hefner KR, Moberg CA, Hachiya LY, Curtin JJ (2013). Alcohol stress response dampening during imminent versus distal, uncertain threat. J Abnorm Psychol.

[139] Little RJ, D'Agostino R, Cohen ML, Dickersin K, Emerson SS, Farrar JT, Frangakis C, Hogan JW, Molenberghs G, Murphy SA, Neaton JD, Rotnitzky A, Scharfstein D, Shih WJ, Siegel JP, Stern H (2012). The prevention and treatment of missing data in clinical trials. N Engl J Med.

[140] Little RJ, Cohen ML, Dickersin K, Emerson SS, Farrar JT, Neaton JD, Shih W, Siegel JP, Stern H (2012). The design and conduct of clinical trials to limit missing data. Stat Med.

[141] McCleary L (2002). Using multiple imputation for analysis of incomplete data in clinical research. Nurs Res.

