# Supplementary material for: Maximizing Engagement, Trust, and Clinical Benefit of AI-Generated Recovery Support Messages for Alcohol Use Disorder: Protocol for an Optimization Study
Source: JMIR Res Protoc. 2025 Nov 7;14:e81697. doi: 10.2196/81697 (PMC12639347; doi:10.2196/81697)
Supplement: Multimedia Appendix 1 [file resprot_v14i1e81697_app1.pdf]

SUMMARY STATEMENT
(Privileged Communication)

PROGRAM CONTACT:
Mariela Shirley
301-402-9389
shirleym@mail.nih.gov

Release Date: **02/28/2024**
Revised Date:

Application Number: **1R01AA031762-01**

Principal Investigator

CURTIN, JOHN J.

Applicant Organization: UNIVERSITY OF WISCONSIN-MADISON

Review Group: **IPTA**
Interventions to Prevent and Treat Addictions Study Section

Meeting Date: **02/15/2024**
Council: **MAY 2024**
Requested Start: **07/01/2024**
Opportunity Number: **PA-20-183**
PCC: **AC S**

Project Title: **Optimizing Smart Digital Therapeutic Message Components for Engagement and Clinical Outcomes for Alcohol Use Disorder**
SRG Action: **Impact Score:31 Percentile:12**
Next Steps: **Visit https://grants.nih.gov/grants/next_steps.htm**
Human Subjects: 48-At time of award, restrictions will apply
Animal Subjects: 10-No live vertebrate animals involved for competing appl.
Gender: 1A-Both genders, scientifically acceptable
Minority: 1A-Minorities and non-minorities, scientifically acceptable
Age: 1A-Children, Adults, Older Adults, scientifically acceptable

Project Year	Direct Costs Requested	Estimated Total Cost
1	481,973	746,918
2	475,907	737,517
3	480,973	745,368
4	481,091	745,551
5	481,928	746,848
TOTAL	2,401,872	3,722,202

ADMINISTRATIVE BUDGET NOTE: The budget shown is the requested budget and has not been adjusted to reflect any recommendations made by reviewers. If an award is planned, the costs will be calculated by Institute grants management staff based on the recommendations outlined below in the COMMITTEE BUDGET RECOMMENDATIONS section.

1R01AA031762-01 Curtin, John

PROTECTION OF HUMAN SUBJECTS: UNACCEPTABLE

RESUME AND SUMMARY OF DISCUSSION: This application proposes to develop a machine learning guided engagement message system that can be added to any smart digital therapeutic to increase risk-relevant engagement and improve clinical outcomes for alcohol use disorder (AUD). The project's high significance lies in its ability to advance what is known about the role of different modalities of messages to increase effective engagement with digital therapeutics for AUD. The investigative team was considered excellent and possessing the necessary expertise related to content areas and methodologies, with an impressive track record and with access to a strong and appropriate research environment, bringing together multiple centers. However, a minor weakness was identified around limited expertise in MOST designs since the only person with this expertise is a consultant rather than investigator. The project's innovation lies in the integration of AI and digital therapeutics to improve key engagement features that might be directly linked to AUD treatment efficacy, with the potential to generate the knowledge base for the SUD and behavioral health field more broadly. The panel was highly enthusiastic about the many strengths of the application, including: the contextualized geolocation approach, the study design based on the MOST framework, the collection of passive data on engagement with the app, as well as the incorporation of the already developed by this team lapse prediction models. Other major strengths included the intervention addressing several components at once, which could increase engagement. The panel also noted some minor and addressable weaknesses in the approach, including around the scientific premise, with insufficient scientific evidence provided in support of the assumption that lack of engagement is about not knowing when or how to use the app, as well as around unclear feasibility of recruitment and insufficiently addressed perceived transparency and trust, which are critical to this application. Concerns were raised around insufficient justification for why informing people of lapse likelihood or likelihood trajectory may be helpful (and not harmful). Relatedly, concerns around human subjects protections were raised due to the potential iatrogenic effects of the intervention. Following the discussion, the panel agreed that the weaknesses identified were minor and addressable and did not detract from the project's high impact. Overall, the proposed project was seen as potentially very important and with high probability of success. Findings are expected to have a high overall impact on the field of precision medicine for alcohol use disorder.

DESCRIPTION (provided by applicant): Clinician-delivered relapse prevention interventions for alcohol use disorder (AUD) are effective when delivered but the vast majority of adults with an active AUD do not receive them due to well-known barriers to clinician-delivered treatment. Digital therapeutics (smartphone "apps" that are used to prevent, treat, or manage a medical or psychiatric disorder) can address these barriers. Unfortunately, the benefits from digital therapeutics may be constrained because engagement with them is often not sustained or matched to patients' needs. The next wave of "smart" digital therapeutics that include embedded machine learning lapse prediction models powered by personal sensing can address these constraints by guiding patients to sustain engagement with the specific interventions and supports that are most personally risk-relevant and therefore most effective. Personal sensing has been possible within digital therapeutics for AUD for several or more years. Machine learning lapse prediction models are emerging now, and the models developed by our team meet or exceed performance thresholds necessary for useful clinical applications. We are well-positioned to develop a smart version of our Center's A-CHESS digital therapeutic by embedding our lapse prediction model into an existing version of our digital therapeutic that already has sensing capabilities. However, we must first determine how best to provide model

feedback to patients so that they use this information and follow its recommendations. In this application, we propose to optimize feedback from our lapse prediction model (via daily engagement messages) both to increase risk-relevant engagement with Smart A-CHESS and to improve clinical outcomes over six months among 416 participants with moderate to severe AUD. Following the Multiphase Optimization Strategy, we factorially manipulate four candidate components of these daily engagement messages that convey transparent, individualized, risk-relevant information from our machine learning lapse prediction model to participants. These message components include: 1) lapse probability, 2) lapse probability change, 3) important model features, and 4) a risk-relevant module recommendation. These components use output that would be available from any machine learning lapse prediction model such that conclusions about the impact of these components on engagement can generalize beyond our specific machine learning model. Similarly, engagement messages including these message components could be used in any smart digital therapeutic for AUD, allowing conclusions to generalize to current and future variants of smart digital therapeutics for AUD. At the conclusion of the grant period, we will also deliver this optimized smart digital therapeutic as a tangible product and model for how to embed sensing and machine learning into other existing digital therapeutics.

PUBLIC HEALTH RELEVANCE: Digital therapeutics for alcohol use disorder are modestly effective but their benefits may be constrained because engagement is often not sustained or matched to patients' needs. "Smart" digital therapeutics that are enhanced by personal sensing and embedded machine learning lapse prediction models that provide individualized, temporally-precise, risk-relevant information to patients may address these limitations. Our broad goal is to develop a machine learning guided engagement message system that can be added to any smart digital therapeutic to increase risk-relevant engagement and improve clinical outcomes.

CRITIQUE 1

Significance: 3
Investigator(s): 1
Innovation: 4
Approach: 4
Environment: 1

Overall Impact: This MOST study could potentially generate an important knowledge base to inform future use and optimization of digital therapeutics for AUD with machine learning and artificial intelligence. Specifically, this study can potentially ascertain the role of different modalities of messages to increase effective engagement with digital therapeutics (e.g., daily lapse probability). The study also aims to increase trust and transparency in these systems. The systematic evaluation of different messaging systems and how are they linked to process and outcomes is innovative. In addition, the integration of AI with an evidence-based digital therapeutic for alcohol use is novel. However, the approach to evaluate this system has some key flaws. Specifically, the lack of qualitative or quantitative measures to rigorously evaluate the experience of patients exposed to each of the messaging manipulations. Considering that the proposal puts a strong emphasis in perceived trust and transparency of AI tools, the lack of a measurement component to evaluate this component of the intervention is a major weakness. The investigators have an extensive track record of research in each of the key components of this proposal (A-CHESS, ML, geolocation, AUD, MOST designs, messaging), and the environment is also equipped to address their stated aims. Overall, this is a proposal with high likelihood of making a significant impact in the field. However, key concerns about their stated purpose and approach around transparency and trust moderate its potential impact.

1. Significance:

Strengths

- The proposal tackles recovery from alcohol use disorder, a key problem with significant impact
- It argues that treatment engagement and lack of scalable solutions is the key barrier, and that the use of digital therapeutics equipped with personal sensing technologies and machine learning approaches to risk relapse detection is the solution.
- It’s important to identify when an individual might relapse or may have a relapse vulnerability. This could increase the effectiveness of digital therapeutics.
- The authors support the importance of developing machine learning models that are transparent and trusty.
- This project is significant because of its potential application to a growing number of digital therapeutics. The development of a new knowledge base around this integration can have a transversal impact on existing digital therapeutics.
- This trial can potentially generate important mechanistic and process knowledge that can improve our understanding of digital therapeutics’ use and efficacy.

Weaknesses

- The reasons for lack of engagement are assumed to be that patients don’t know when to use an app, and which modules are best for them. But the causal link between the two is yet to be proven or justified.
- Existing studies showing a causal link between engagement with a digital therapeutic and clinical outcomes are missing.
- In the absence of a mixed-methods or more direct qualitative component, it’s unclear how trust in AI systems will be rigorously evaluated. A MOST design narrowly focused on a few manipulating variables cannot provide insight into those factors. Either a review of the qualitative literature on those factors, or a more exploratory approach to this question is missing.

2. Investigator(s):

Strengths

- The PI has an impressive track record of NIH funded research in areas directly relevant to the successful completion of the proposed project.
- The PI has extensive experience conducing quantitative analyses of alcohol, SUD, passive sensing and EMA data, including machine learning.
- Extensive range of expertise in Co-Is and consultants on relevant areas needed for this proposal, including the developer or A-CHESS (Gustafson), expert in geolocation ML systems (Mohr), MOST designs (Murphy and Collins).

Weaknesses

- None noted by reviewer.

3. Innovation:

Strengths

- This study aims to integrate AI and digital therapeutics to improve key engagement features that might be directly linked to treatment efficacy.
- The results of this study may generate the base knowledge for future integration of AI with other DTx in the SUD and behavioral health field.

Weaknesses

- Addressing trust and transparency seems an innovation, but it is unclear how it will be addressed and measured in this study proposal and how it will rigorously inform the field.

4. Approach:

Strengths

- The contextualized geolocation approach, combining passive sensing with EMA confirmation and categorization is rigorous and has been validated in prior research.
- The aims of the grant are largely independent of the specific digital therapeutic being used to test the AI system. However, utilizing CHESS as the base platform is an ideal start point.
- Collecting EMA data only once per day seems feasible and will mitigate assessment burden.
- The team has an extensive track record developing the accuracy and use of geolocation in digital interventions.
- The machine learning model has already been trained and tested in prior research among AUD populations with high levels of specificity and sensitivity.
- The use of the SHAP method is in line with the relapse prevention model, helping users identify most optimal interventions and supports.
- The notifications and message schedule has been carefully considered and refined to avoid disruptions and increase engagement and uptake.
- A pool of core statements based on CoI Van Swol on empathy, feasibility and clarity in health advice is a significant strength.
- The use of the user engagement scale and the digital therapeutic alliance add rigor to the measurement approach and provide a new context to interpret the objective measures of engagement.
- Well justified use of the primary outcomes for AUD based on FDA guidelines, including TLFB and EMA reporting.

Weaknesses

- The key manipulation is designed to make various aspects of the lapse prediction model more transparent to the participant. However, it is unclear how perceived transparency will be measured rigorously in this study in the absence of qualitative process measures. An initial focus on treatment engagement and clinical outcomes is necessary (aims 1 and 2), but not sufficient.
- It’s unclear what percentage of the available smartphones in the market will be able to support A-CHESS. Without more information about the technical requirements of A-CHESS it’s hard to evaluate its potential use. Minor.
- It’s unclear which medical or psychiatric co-morbidities are expected to preclude smartphone use. The PHQ-9 and the GAD-7 will only capture a very limited range of psychiatric symptoms.

- Categorizing EMAs as ´sensing´ is misleading. Based on that definition, any questionnaire, self-report measure, or behavioral observation could be categorized as ‘sensing’. Describing them as ‘active sensing’ does not resolve the concern with assessment burden, or accuracy of reporting. The goal of reporting 1x EMA per day mitigates this concern, but the use of an incentive to complete the EMAs adds the concern of its implementation potential (from a payer’s perspective) of this approach in real world settings. Moderate.
- Providing monetary compensation for EMA completion reduces the ‘implementability’ of this intervention in real world settings. This implication has not been discussed. Moderate.
- Lack of details about the size of the pool of statements and message content to be used in this study. Minor

5. Environment:

Strengths

- The Center for Health Enhancement and Systems Studies developed the CHESS digital therapeutic and offers an excellent context to implement the AI based system planned in this project.
- The Addiction Research Center directed by Dr. Curtin, offers extensive expertise and support to conduct AUD research.
- The center for high throughput computing offers ML and AI expertise to support this proposal.
- Both environment and investigators line up in terms of history of collaboration and leadership at these centers at UWM

Weaknesses

- None noted by reviewer.

Study Timeline:

Strengths

- The timeline seems feasible.

Weaknesses

- None noted by reviewer.

Protections for Human Subjects:

Acceptable Risks and/or Adequate Protections

Data and Safety Monitoring Plan (Applicable for Clinical Trials Only):

Acceptable

Inclusion Plans:

- Sex/Gender: Distribution justified scientifically
- Race/Ethnicity: Distribution justified scientifically
- For NIH-Defined Phase III trials, Plans for valid design and analysis: Scientifically acceptable
- Inclusion/Exclusion Based on Age: Distribution justified scientifically

Vertebrate Animals:

Not Applicable (No Vertebrate Animals)

Biohazards:

Not Applicable (No Biohazards)

Resource Sharing Plans:

Acceptable

- Not available

Budget and Period of Support:

Recommend as Requested

CRITIQUE 2

Significance: 2
Investigator(s): 2
Innovation: 1
Approach: 3
Environment: 1

Overall Impact: This proposal is designed to optimize engagement and clinical outcomes of a digital therapeutic for alcohol use disorder, by manipulating four message components in a Multiphase Optimization Strategy Trial. There are several exciting aspects to the proposed work, beginning with an ideal interdisciplinary team with extensive expertise in all areas relevant to the proposal. Significance is high, in that not only can the use of personal sensing and machine learning enhance capabilities of the digital intervention tested here (A-CHESS), but the applicants propose to deliver a model for embedding these features into any existing digital therapeutic. The proposed work is extremely cutting-edge and can take A-CHESS (and other digital therapies) to the next level if successful. There are also many design strengths, including the MOST design to efficiently test 4 message components, the ability to passively sense one of the outcomes (app engagement), and use of existing lapse prediction models developed by this team. This reviewer’s most major critiques include little justification for why informing people of lapse likelihood or likelihood trajectory may be helpful (and not harmful) and concerns about feasibility of recruitment. Nonetheless, given its high innovation and significance, and the strong team, this proposal is overall deemed outstanding.

1. Significance:

Strengths

- Personal sensing and use of machine learning can greatly enhance the capabilities of digital interventions, and the PI has used such methods to detect alcohol lapse likelihood. Digital interventions have potential to reduce health disparities in delivery of AUD treatments.
- Determining which intervention message components increase engagement and improve clinical outcomes is a strength.

- The proposal has a basis in relapse prevention models, which have much prior empirical support. This, combined with a high level of planned personalization of intervention content, set the stage for an especially effective digital intervention.
- The team proposes to deliver a model for embedding sensing and machine learning into digital therapeutics other than the single one studied here.

Weaknesses

- The rationale for each of the four message components tested (why each may uniquely improve outcomes, particularly informing one of their lapse probability and probability change) is underdeveloped.
- While a smart digital therapy will be optimized (a great strength), it will not be able to also be evaluated in the study timeframe. In other words, this work will not reveal whether the Smart A-CHESS is any better than the existing A-CHESS. This is a minor concern, given that optimization must occur first.

2. Investigator(s):

Strengths

- PI Curtin has an extensive history of involvement in longitudinal studies and RCTs for substance use disorders. He also has expertise in passive sensing and data analytic techniques relevant to the proposed work. An additional strength of the PI is that he is a clinical psychologist with expertise in the treatment of substance use disorders.
- Co-I Gustafson is an industrial engineer with 40+ years of experience developing and testing technologies and theory-based models to improve human health, including the A-CHESS digital therapeutic that is the focus of this application.
- Co-I Mohr also brings expertise in behavioral science, clinical interventions and technology, including the use of mobile phone sensors to identify behavioral phenotypes.
- Co-I Kornfield is a health communication researcher currently supported by a K award, with expertise in digital health and user-centered design and contributes prior work testing A-CHESS.
- Co-I Van Swol is a social psychologist with unique expertise in message factors that increase likelihood of advice perception and uptake.
- Co-I Ammerman has experience in intensive longitudinal methods, passive sensing, and development of smartphone interventions.
- Consultant Murphy is a recognized expert and leading developer of microrandomized trials and the MOST framework.
- The team has a nice mix of established and earlier-career researchers, from multiple disciplines.

Weaknesses

- Some on the team have not previously collaborated. Co-I Van Swol does not appear to have previously collaborated with others on this team (and it is unclear if they have collaborated on any other grant-funded teams). There is no evidence that Co-I Ammerman has previously collaborated with others listed, and unique contribution of this collaborator is not entirely clear. This concern is minor given the unique expertise Van Swol contributes and the strength of the interdisciplinary team overall.

- Only the consultant, and not investigators, have expertise in MOST, a key component of the study design.

3. Innovation:

Strengths

- For the first time, A-CHESS will be enhanced by messaging that is extremely personal to the user (based on their passively sensed data) and that can prompt use of ideal intervention components.
- Adaptations to intervention guided by sensing and machine learning is cutting-edge.
- A novel component of this study involves the focus of human-machine communications in advice messaging.

Weaknesses

- None noted by reviewer.

4. Approach:

Strengths

- A MOST design allows manipulation of several message components that may increase intervention engagement and intervention outcomes.
- A-CHESS is a justifiable choice for the intervention to optimize, as a starting point.
- Engagement with the app can be passively sensed.
- EMAs are delivered at a participant-selected time, and PI Curtin has achieved high EMA compliance in prior work.
- Lapse prediction models already developed by the team can be incorporated here.
- The messages will be designed to prioritize new information/suggestions, which will likely help to maintain engagement.
- Outcome variables are well-justified and well-measured.

Weaknesses

- Recruitment will occur via social media. This makes verification of AUD and initial remission difficult, relying only on self-report. Further, given the recent landscape of online recruitment, methods for detecting and diminishing scam participants (e.g., those outside the US) are lacking.
- Recruitment of 15 participants per month who meet quite strict eligibility criteria (at least moderate AUD, in initial remission) seems ambitious, particularly without provision of data on how many are expected to need to complete the intake.
- Lapse probability is quantified as a linear function; whether it is indeed linear likely differs from person to person and over time (minor concern).
- Payment for EMAs, which is conducted through the A-CHESS app, as well as for location sharing, may result in biased rates of usage of the program. While this concern is minor given that usage of the app will be necessary to address primary aims, likely dissemination/implementation of the program will be unclear at the conclusion of the study.

- There may be a missed opportunity to collect qualitative data from participants on what worked/did not work well for them regarding the engagement messages.
- Evidence that it is ideal to send engagement messages overnight, for viewing in the morning, is not presented. It seems that lapse prediction and need for intervention may be more likely later in the day, rather than (presumably) being based on passively sensed data from the prior day.

5. Environment:

Strengths

- The Center for Health Enhancement Systems Studies, Addiction Research Center, and Center for Highthroughput Computing at University of Wisconsin provide an excellent environment to support the proposed work. The strengths of the Wisconsin site are supplemented by the strengths of Northwestern University, where two of the co-Is are based.

Weaknesses

- None noted by reviewer.

Study Timeline:

Strengths

- Ample time is planned for study start up and dissemination of results.

Weaknesses

- Recruitment of 15 participants per month may be overly ambitious.

Protections for Human Subjects:

Unacceptable Risks and/or Inadequate Protections

- It seems possible that informing individuals that they are at high likelihood for lapse could have iatrogenic effects. Evidence that informing one of lapse likelihood is helpful rather than harmful would assuage this concern. If unknown, human support in the context of likely lapse might be considered.

Data and Safety Monitoring Plan (Applicable for Clinical Trials Only):

Acceptable

- Scheduled monthly meetings led by PI.

Inclusion Plans:

- Sex/Gender: Distribution justified scientifically
- Race/Ethnicity: Distribution justified scientifically
- Inclusion/Exclusion Based on Age: Distribution justified scientifically
- Plans to recruit equal numbers of males and females, and to slightly oversample for some racial/ethnic minority groups.

Vertebrate Animals:

Not Applicable (No Vertebrate Animals)

Biohazards:

Not Applicable (No Biohazards)

Resource Sharing Plans:

Acceptable

- A detailed data sharing plan that outlines what we be shared and where is not included in the application.

Budget and Period of Support:

Budget Modifications Recommended (in amount/time)
Recommended budget modifications or possible overlap identified:

- $10K is budgeted for recruitment in Year 5, even though data collection is proposed to end in Year 4.

CRITIQUE 3

Significance: 1
Investigator(s): 1
Innovation: 1
Approach: 2
Environment: 1

Overall Impact: This new R01 proposes to use machine learning models to predict alcohol lapse to tailor content of A-CHESS, a popular app for reducing alcohol lapse among individuals recovering from alcohol use disorder, by directing users to the most risk-relevant content. Precision medicine has great potential to improve clinical outcomes for individuals recovering from alcohol use disorder, especially for cost-effective digital therapeutics. Further, the proposed research will improve the technological capability to provide precision medicine for alcohol use disorder. The proposed research is thoughtfully designed to adhere to the MOST framework for evaluating digital therapeutics. The assembled investigative team are at the forefront of research on machine learning and digital therapeutics for substance use. In sum, the proposed research has high potential to have a sustained, powerful influence on precision medicine for alcohol use disorder.

1. Significance:

Strengths

- The use of machine learning to individualize the content of alcohol intervention has great potential to bolster effects to reduce the suffering from harmful alcohol use and alcohol use disorder.
- The proposed research will improve the technical capability for precision medicine for A-CHESS and other mobile interventions.

- The extant literature provides robust support for A-CHESS and recent studies conducted by the investigative team suggest strong feasibility for using a machine learning prediction model of alcohol lapse to successfully develop Smart A-CHESS.

Weaknesses

- None noted by reviewer.

2. Investigator(s):

Strengths

- The assembled investigative team is comprised of experts from three leading centers on digital therapeutics with complementary expertise to conduct the proposed research.
- The investigative team has a strong history of successful collaborations.
- The investigative team has conducted recent studies that provide a foundation for and demonstrate the feasibility of the proposed research.

Weaknesses

- None noted by reviewer.

3. Innovation:

Strengths

- Incorporating machine learning models of alcohol lapse to deliver personalize components of A-CHESS with broader implications for other digital therapeutics is highly innovative.

Weaknesses

- None noted by reviewer.

4. Approach:

Strengths

- Prediction model features grounded in the Relapse Prevention model will be randomly assigned.
- Study design based on the MOST framework to evaluate smart digital therapeutics.
- Alcohol use assessed and verified using both EMA and TLFB.
- Passive collection of geolocation data will be collected which can result in further optimization.

Weaknesses

- Design decisions were well justified to adhere to the MOST framework, but it was somewhat unclear what the benefits of randomization at the participant level, as opposed to a more micro-randomization approach (e.g., daily level), are at this stage.

5. Environment:

Strengths

- The environment is well suited to conduct the proposed research.

Weaknesses

- None noted by reviewer.

Study Timeline:

Strengths

- Timeline is sufficient and well justified.

Weaknesses

- None noted by reviewer.

Protections for Human Subjects:

Acceptable Risks and/or Adequate Protections

- The primary risk is breach of confidentiality, and several safeguards are proposed to minimize its potential of occurring.

Data and Safety Monitoring Plan (Applicable for Clinical Trials Only):

Acceptable

- Participants will adhere to NIH and institutional IRB standards for reporting adverse events and PI will monitor safety, data accuracy, and confidentiality.

Inclusion Plans:

- Sex/Gender: Distribution justified scientifically
- Race/Ethnicity: Distribution justified scientifically
- Inclusion/Exclusion Based on Age: Distribution justified scientifically
- Equal numbers of women and men will be recruited for adequate statistical power for exploratory analyses examining sex differences.
- Participants who identify as racial/ethnic minorities will be over sampled to ensure adequate representation, and exploratory analyses will examine differences across race/ethnicity.
- Exclusion of children is scientifically justified.

Vertebrate Animals:

Not Applicable (No Vertebrate Animals)

Biohazards:

Not Applicable (No Biohazards)

Resource Sharing Plans:

Not Applicable (No Relevant Resources)

Budget and Period of Support:

Recommend as Requested

THE FOLLOWING SECTIONS WERE PREPARED BY THE SCIENTIFIC REVIEW OFFICER TO SUMMARIZE THE OUTCOME OF DISCUSSIONS OF THE REVIEW COMMITTEE, OR REVIEWERS' WRITTEN CRITIQUES, ON THE FOLLOWING ISSUES:

PROTECTION OF HUMAN SUBJECTS: UNACCEPTABLE. The protection of human subjects from research risks is unacceptable. There are concerns around potential iatrogenic effects of the intervention. This needs to be addressed.

INCLUSION OF WOMEN PLAN: ACCEPTABLE

INCLUSION OF MINORITIES PLAN: ACCEPTABLE

INCLUSION ACROSS THE LIFESPAN: ACCEPTABLE

COMMITTEE BUDGET RECOMMENDATIONS: The budget was recommended as requested.

Footnotes for 1R01AA031762-01; PI Name: Curtin, John J.

NIH has modified its policy regarding the receipt of resubmissions (amended applications).See Guide Notice NOT-OD-18-197 at https://grants.nih.gov/grants/guide/notice-files/NOT-OD-18-197.html. The impact/priority score is calculated after discussion of an application by averaging the overall scores (1-9) given by all voting reviewers on the committee and multiplying by 10. The criterion scores are submitted prior to the meeting by the individual reviewers assigned to an application, and are not discussed specifically at the review meeting or calculated into the overall impact score. Some applications also receive a percentile ranking. For details on the review process, see http://grants.nih.gov/grants/peer_review_process.htm#scoring.

MEETING ROSTER

Interventions to Prevent and Treat Addictions Study Section
Risk, Prevention and Health Behavior Integrated Review Group
CENTER FOR SCIENTIFIC REVIEW
IPTA
02/15/2024 - 02/16/2024

Notice of NIH Policy to All Applicants: Meeting rosters are provided for information purposes only. Applicant investigators and institutional officials must not communicate directly with study section members about an application before or after the review. Failure to observe this policy will create a serious breach of integrity in the peer review process, and may lead to actions outlined in NOT-OD-22-044 at https://grants.nih.gov/grants/guide/notice-files/NOT-OD-22-044.html, including removal of the application from immediate review.

CHAIRPERSON(S)

PIPER, MEGAN E, PHD
PROFESSOR
DEPARTMENT OF MEDICINE
UNIVERSITY OF WISCONSIN-MADISON
MADISON, WI 53711

MEMBERS

AUDRAIN-MCGOVERN, JANET, PHD
PROFESSOR AND DIRECTOR
DEPARTMENT OF PSYCHIATRY
PERELMAN SCHOOL OF MEDICINE
UNIVERSITY OF PENNSYLVANIA
PHILADELPHIA, PA 19104

BRICKER, JONATHAN B, PHD
PROFESSOR
DIVISION OF PUBLIC HEALTH SCIENCES
FRED HUTCHINSON CANCER RESEARCH CENTER
UNIVERSITY OF WASHINGTON
SEATTLE, WA 98109

CLAUS, ERIC D, PHD
ASSOCIATE PROFESSOR
DEPARTMENT OF BIOBEHAVIORAL HEALTH
THE PENNSYLVANIA STATE UNIVERSITY
UNIVERSITY PARK, PA 16802

COMIFORD, ASHLEY, DRPH *
EPIDEMIOLOGIST
DEPARTMENT OF HEALTH SERVICES
CHEROKEE NATION HEALTH SERVICES
TAHLEQUAH, OK 74464

DERMEN, KURT H, PHD *
SENIOR RESEARCH SCIENTIST
DEPARTMENT OF PSYCHIATRY
JACOBS SCHOOL OF MEDICINE AND BIOMEDICAL SCIENCES
UNIVERSITY AT BUFFALO
STATE UNIVERSITY OF NEW YORK
BUFFALO, NY 14203

DRAZDOWSKI, TESS K, PHD *
RESEARCH SCIENTIST & LICENSED PSYCHOLOGIST
LIGHTHOUSE INSTITUTE
CHESNUT HEALTH SYSTEMS
EUGENE, OR 97401

FENDRICH, MICHAEL, PHD *
PROFESSOR
DEPARTMENT OF EMERGENCY MEDICINE
MEDICAL COLLEGE OF WISCONSIN
MILWAUKEE, WI 53226

KONG, GRACE, PHD
ASSOCIATE PROFESSOR
DEPARTMENT OF PSYCHIATRY
YALE UNIVERSITY SCHOOL OF MEDICINE
NEW HAVEN, CT 06519

LEDGERWOOD, DAVID M, PHD
PROFESSOR
DEPARTMENT OF PSYCHIATRY
AND BEHAVIORAL NEUROSCIENCES
SCHOOL OF MEDICINE
WAYNE STATE UNIVERSITY
DETROIT, MI 48201

LEE, DUSTIN CLARK, PHD *
ASSOCIATE PROFESSOR
DEPARTMENT OF PSYCHIATRY AND BEHAVIORAL SCIENCES
THE JOHNS HOPKINS SCHOOL OF MEDICINE
BALTIMORE, MD 21224

MACATEE, RICHARD, PHD *
ASSISTANT PROFESSOR
DEPARTMENT OF PSYCHOLOGICAL SCIENCES
AUBURN UNIVERSITY
AUBURN, AL 36849

MARTINEZ PRADEDA, URSULA, PHD *
ASSISTANT PROFESSOR
DEPARTMENT OF FAMILY AND PREVENTIVE MEDICINE
UNIVERSITY OF UTAH
SALT LAKE CITY, UT 84112

MCCHARGUE, DENNIS E, PHD *
PROFESSOR AND DIRECTOR OF CLINICAL TRAINING
DEPARTMENT OF PSYCHOLOGY
COLLEGE OF ARTS AND SCIENCES
UNIVERSITY OF NEBRASKA-LINCOLN
LINCOLN, NE 68516

MCHUGH, REBECCA KATHRYN, PHD
ASSOCIATE PROFESSOR
DEPARTMENT OF PSYCHIATRY
HARVARD MEDICAL SCHOOL
BELMONT, MA 02478

MCPHERSON, STERLING M, PHD
DIRECTOR AND PROFESSOR
PROGRAM OF EXCELLENCE IN ADDICTIONS RESEARCH
DEPARTMENT OF COMMUNITY AND BEHAVIORAL HEALTH
ELSON S. FLOYD COLLEGE OF MEDICINE
WASHINGTON STATE UNIVERSITY
SPOKANE, WA 99210

MERRILL, JENNIFER ELIZABETH, PHD *
ASSOCIATE PROFESSOR
DEPARTMENT OF BEHAVIORAL AND SOCIAL SCIENCES
BROWN UNIVERSITY
PROVIDENCE, RI 02912

MINAMI, HARUKA, PHD *
ASSOCIATE PROFESSOR
DEPARTMENT OF PSYCHOLOGY
FORDHAM UNIVERSITY
THE BRONX, NY 10458

MONTGOMERY, LATRICE, PHD
DIRECTOR OF CLINICAL RESEARCH AT RIA HEALTH,
ADJUNCT ASSOCIATE PROFESSOR
DEPARTMENT OF PSYCHIATRY AND
BEHAVIORAL NEUROSCIENCE
COLLEGE OF MEDICINE
UNIVERSITY OF CINCINNATI
CINCINNATI, OH 45229

MUMBA, MERCY N, PHD
ASSOCIATE PROFESSOR
CAPSTONE COLLEGE OF NURSING
THE UNIVERSITY OF ALABAMA
TUSCALOOSA, AL 35401

OSTACHER, MICHAEL JOSHUA, MD *
PROFESSOR
DEPARTMENTS OF PSYCHIATRY AND BEHAVIORAL
SCIENCES
STANFORD UNIVERSITY
PALO ALTO, CA 94304

PANG, RAINA, PHD *
ASSOCIATE PROFESSOR
DEPARTMENT POPULATION AND PUBLIC SCIENCES
KECK SCHOOL OF MEDICINE
UNIVERSITY OF SOUTHERN CALIFORNIA
LOS ANGELES, CA 90033

RICHARDS, DYLAN K, PHD *
ASSISTANT PROFESSOR
CENTER ON ALCOHOL, SUBSTANCE USE, AND ADDICTIONS
UNIVERSITY OF NEW MEXICO
ALBUQUERQUE, NM 87106

SADASIVAM, RAJANI, PHD
PROFESSOR
DIVISION OF HEALTH AND IMPLEMENTATION SCIENCE
DEPARTMENT OF POPULATION AND QUANTITATIVE
HEALTH SCIENCES
UNIVERSITY OF MASSACHUSETTS MEDICAL SCHOOL
WORCESTER, MA 01605

SCHACHT, REBECCA L, PHD *
ASSISTANT PROFESSOR
DEPARTMENT OF PSYCHOLOGY
UNIVERSITY OF MARYLAND BALTIMORE COUNTY
BALTIMORE, MD 21250

SCHMITZ, JOY MARIE, PHD
PROFESSOR
DEPARTMENT OF PSYCHIATRY AND BEHAVIORAL
SCIENCES
UNIVERSITY OF TEXAS HEALTH SCIENCE CENTER
HOUSTON, TX 77054

SCOTT, KELLI, PHD *
ASSISTANT PROFESSOR
DEPARTMENT OF MEDICAL SOCIAL SCIENCES
NORTHWESTERN UNIVERSITY
CHICAGO, IL 60613

SPEARS, CLAIRE ADAMS, PHD
ASSOCIATE PROFESSOR
DEPARTMENT OF HEALTH POLICY & BEHAVIORAL
SCIENCES
SCHOOL OF PUBLIC HEALTH
GEORGIA STATE UNIVERSITY
ATLANTA, GA 30303

SPILLANE, NICHEA SOLOMON, PHD
ASSOCIATE PROFESSOR
DEPARTMENT OF PSYCHOLOGY
COLLEGE OF HEALTH SCIENCES
UNIVERSITY OF RHODE ISLAND
KINGSTON, RI 02881

STEVENS-WATKINS, DANELLE J, PHD
PROFESSOR
DEPARTMENT OF EDUCATIONAL, SCHOOL, AND COUNSELING PSYCHOLOGY
COLLEGE OF EDUCATION
UNIVERSITY OF KENTUCKY
LEXINGTON, KY 40506

STORMSHAK, ELIZABETH A, PHD
PROFESSOR
COLLEGE OF EDUCATION
PREVENTION SCIENCE INSTITUTE
UNIVERSITY OF OREGON
EUGENE, OR 97403

VILARDAGA, ROGER, PHD
ASSOCIATE PROFESSOR
DEPARTMENT OF PSYCHIATRY AND BEHAVIORAL SCIENCES
SCHOOL OF MEDICINE
DUKE UNIVERSITY
DURHAM, NC 27710

WALLEY, ALEXANDER YALE, MD *
PROFESSOR
CLINICAL ADDICTION RESEARCH AND EDUCATION UNIT
SECTION OF GENERAL INTERNAL MEDICINE
GRAYKEN CENTER FOR ADDICTION BOSTON MEDICAL CENTER
BOSTON UNIVERSITY
BOSTON, MA 02118

WARD, MELISSA KARI, PHD *
ASSISTANT PROFESSOR
DEPARTMENT OF EPIDEMIOLOGY
FLORIDA INTERNATIONAL UNIVERSITY
MIAMI, FL 33199

YI, RICHARD, PHD
PROFESSOR AND DIRECTOR
COFRIN LOGAN CENTER FOR ADDICTION RESEARCH AND TREATMENT
DEPARTMENT OF PSYCHOLOGY
UNIVERSITY OF KANSAS
LAWRENCE, KS 66045

SCIENTIFIC REVIEW OFFICER

ZANDBERG, IZABELLA, PHD
SCIENTIFIC REVIEW OFFICER
CENTER FOR SCIENTIFIC REVIEW
BETHESDA, MD 20892

EXTRAMURAL SUPPORT ASSISTANT

MACPHERSON, SETH LOPAKI
EXTRAMURAL SUPPORT ASSISTANT
CENTER FOR SCIENTIFIC REVIEW
NATIONAL INSTITUTES OF HEALTH
BETHESDA, MD 20892

* Temporary Member. For grant applications, temporary members may participate in the entire meeting or may review only selected applications as needed.

Consultants are required to absent themselves from the room during the review of any application if their presence would constitute or appear to constitute a conflict of interest.
